# A Biomimetic cRGDyk‐Engineered Platelet Nanoplatform for ACAD11 Delivery to Trigger Pyroptosis in Hepatocellular Carcinoma

**DOI:** 10.1002/advs.78219

**Published:** 2026-10-08

**Authors:** Hongyi Bao, Wei Feng, Wei Zong, Yi Li, Xinyue Qin, Xinyue Wang, Yubo Yang, Guanhua Chen, Yiting Hou, Xuan Tan, Lin Chen, Ming Zhang, Feng Wang

**Affiliations:** ^1^ Department of Laboratory Medicine Wuxi Medical Center The Affiliated Wuxi People's Hospital of Nanjing Medical University Wuxi People's Hospital Nanjing Medical University Wuxi China; ^2^ Research Center of Clinical Medicine Affiliated Hospital of Nantong University Medical School of Nantong University Nantong China; ^3^ Nantong Institute of Liver Diseases Nantong Third People's Hospital Affiliated Nantong Hospital 3 of Nantong University Nantong China; ^4^ Department of Laboratory Medicine Changzhou Maternal and Child Health Care Hospital Changzhou China

**Keywords:** ACAD11, active targeting, bioengineered platelets, hepatocellular carcinoma, pyroptosis

## Abstract

The therapeutic efficacy against hepatocellular carcinoma (HCC) is frequently compromised by inherent apoptotic evasion and a hostile tumor microenvironment (TME). Inducing immunogenic cell death, particularly pyroptosis, represents a promising avenue to breach these barriers. Herein, we identify acyl‐CoA dehydrogenase family member 11 (ACAD11) as a pivotal metabolic regulator that drives the ROS/NLRP3/GSDMD pyroptotic signaling axis. To therapeutically exploit this mechanism, we engineered a biomimetic delivery nanoplatform comprising cRGDyk‐decorated platelets loaded with ACAD11‐overexpressing plasmids (OE‐ACAD11@R‐PLT). By synergizing the intrinsic tumor‐homing capabilities of platelets with cRGDyk‐mediated active targeting, this system ensures precise accumulation in HCC tissues and facilitates efficient intracellular payload internalization. Upon cytosolic delivery, restored ACAD11 triggers severe mitochondrial dysfunction and subsequent GSDMD‐executed pyroptosis. This lytic cell death releases a surge of damage‐associated molecular patterns (DAMPs), effectively remodeling the immunosuppressive TME into a highly immunogenic and pro‐inflammatory milieu, thereby provoking a systemic anti‐tumor immunity. Collectively, this work establishes a mechanism‐driven, bioengineered strategy that leverages metabolic reprogramming to overcome therapeutic resistance, offering a robust paradigm for next‐generation HCC nanomedicine.

## Graphical abstract

A biomimetic platelet nanoplatform (OE‐ACAD11@R‐PLT) is developed for targeted hepatocellular carcinoma immunotherapy. By selectively delivering therapeutic plasmids, this system induces mitochondrial dysfunction and triggers robust NLRP3/Caspase‐1/GSDMD‐mediated pyroptosis in tumor cells. The subsequent release of tumor antigens and inflammatory cytokines promotes dendritic cell maturation and T cell activation, achieving highly efficient immune‐mediated tumor elimination.

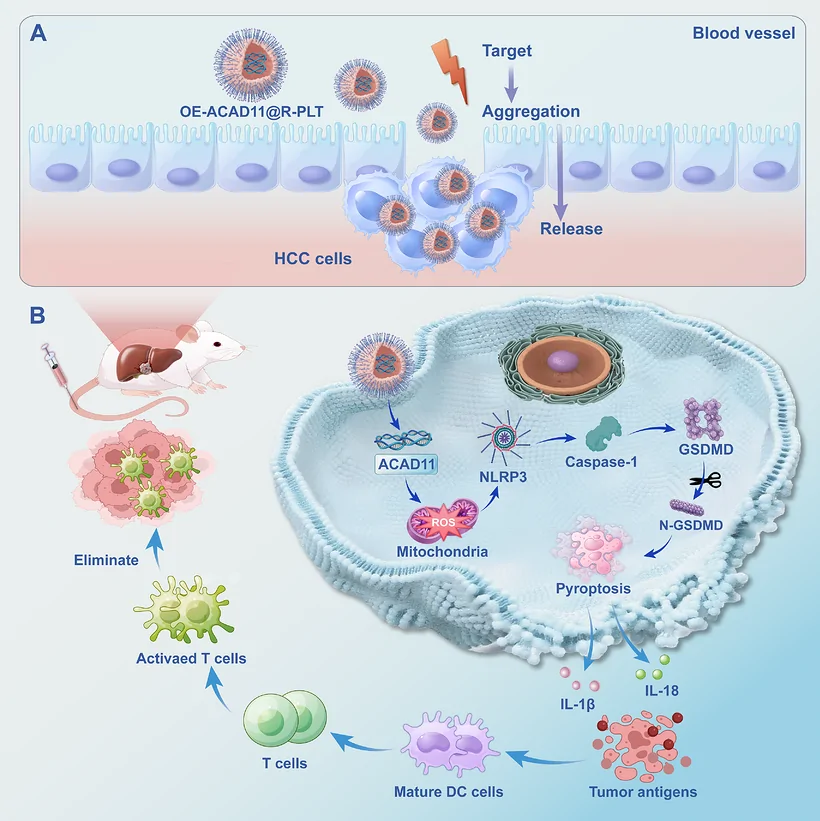

## 1 Introduction

Hepatocellular carcinoma is the predominant proportion of primary hepatic malignancies and serves as a major contributor to the global burden of cancer‐associated death [1, 2, 3]. Characterized by rapid progression and a highly immunosuppressive tumor microenvironment, HCC typically exhibits a poor response to conventional therapeutic modalities, such as chemotherapy and immune checkpoint blockade (ICB) [1, 4]. Pyroptosis is capable of achieving the lytic eradication of chemotherapy‐resistant cells while simultaneously releasing potent immunostimulatory signals [5]. Consequently, the development of therapeutic strategies that drive pyroptosis necessitates the exploration of novel approaches to overcome apoptosis resistance, thereby ensuring precise tumor elimination and the concurrent activation of anti‐tumor immunity [6, 7].

Pyroptosis, a gasdermin‐mediated inflammatory cell death pathway, has recently garnered significant attention as a promising therapeutic avenue in cancer treatment [8]. Unlike apoptosis, which exhibits immunological silence, pyroptosis is characterized by a rupture in the cell membrane, accompanied by the massive efflux of pro‐inflammatory cytokines (e.g., IL‐1β and IL‐18) and tumor‐associated antigens [9]. This process triggers a robust inflammatory cascade, effectively transforming “cold,” immunosuppressive tumors into “hot” environments susceptible to immunological attack [10]. Recent advances in nanomedicine have further demonstrated the potential of nano‐enabled strategies to induce tumor cell pyroptosis and enhance antitumor immunity. For instance, ultrasound‐responsive nanoplatforms have been developed to amplify oxidative stress and activate pyroptotic signaling, thereby promoting immunogenic tumor destruction and strengthening antitumor immune responses [11, 12]. In addition, engineered magnetic nano–micro systems integrating pyroptosis induction with locoregional therapy have shown considerable potential for remodeling the immunosuppressive tumor microenvironment and potentiating cancer immunotherapy [13]. The NLRP3 inflammasome acts as a critical sensor in this cascade, activating Caspase‐1 (CASP‐1) to cleave Gasdermin D (GSDMD) [14, 15]. However, in the context of HCC, the specific upstream regulators capable of precisely activating NLRP3‐mediated pyroptosis remain unclear. In this study, we identified Acyl‐CoA Dehydrogenase Family Member 11 (ACAD11) as a potent activator of the NLRP3 inflammasome.

Despite the identification of the promising therapeutic target, the clinical translation of gene therapy is frequently impeded by delivery barriers [16]. Traditional viral vectors, such as adenoviruses or lentiviruses, while offering high transfection efficiency, are compromised by significant safety concerns, including robust host immune responses and potential genotoxicity arising from insertional mutagenesis [17]. While offering superior biosafety, synthetic vectors are limited by physiological instability and poor targeting, resulting in rapid clearance and low tumor retention [18, 19]. To overcome these limitations, platelets (PLTs) have garnered significant attention as a natural delivery vehicle attributable to their favorable biocompatibility and innate capability of homing toward damaged blood vessels and sites of inflammation [20, 21].

Within the specific pathological landscape of HCC therapy, this natural inflammatory tropism poses a formidable challenge [22]. Given that HCC patients often harbour underlying chronic hepatitis or cirrhosis, native platelets are susceptible to non‐specific retention due to the pervasive inflammatory background within the liver, thereby limiting their effective accumulation within the tumor core [23, 24]. To address this issue, we employed a surface modification strategy using the cRGDyk peptide. The cRGDyk peptide exhibits a high affinity for integrin α_v_β_3_, a receptor significantly overexpressed on tumor neovasculature and tumor cells but minimally expressed in normal liver tissues [25, 26]. This differential expression pattern empowers the cRGDyk peptide with unique active targeting capabilities against HCC [27].

In this study, a biomimetic precision therapy system (OE‐ACAD11@R‐PLT) was fabricated based on the essential role of ACAD11. This system integrates cRGDyk peptide‐modified platelets loaded with ACAD11‐overexpressing plasmids to potentiate targeted delivery to HCC cells. Through the specific recognition of integrin α_v_β_3_ by cRGDyk, OE‐ACAD11@R‐PLT is able to evade non‐specific sequestration by the reticuloendothelial system and achieve high‐efficiency enrichment at the tumor site (Scheme 1A). This accumulation facilitates the intracellular release of the gene vector, wherein the upregulation of ACAD11 triggers a mitochondrial reactive oxygen species (ROS) burst. Subsequent activation of the NLRP3/Caspase‐1 axis by this oxidative stress culminates in GSDMD cleavage and the execution of tumor cell pyroptosis. Following pyroptosis, the release of pro‐inflammatory cytokines (IL‐1β, IL‐18) and tumor antigens promotes dendritic cell maturation and recruits activated T cells, thereby remodeling the immunosuppressive tumor microenvironment (Scheme 1B). This approach presents an engineered biomimetic system for targeted HCC gene therapy, expanding the therapeutic promise of pyroptosis‐mediated immunotherapy.

**SCHEME 1:**
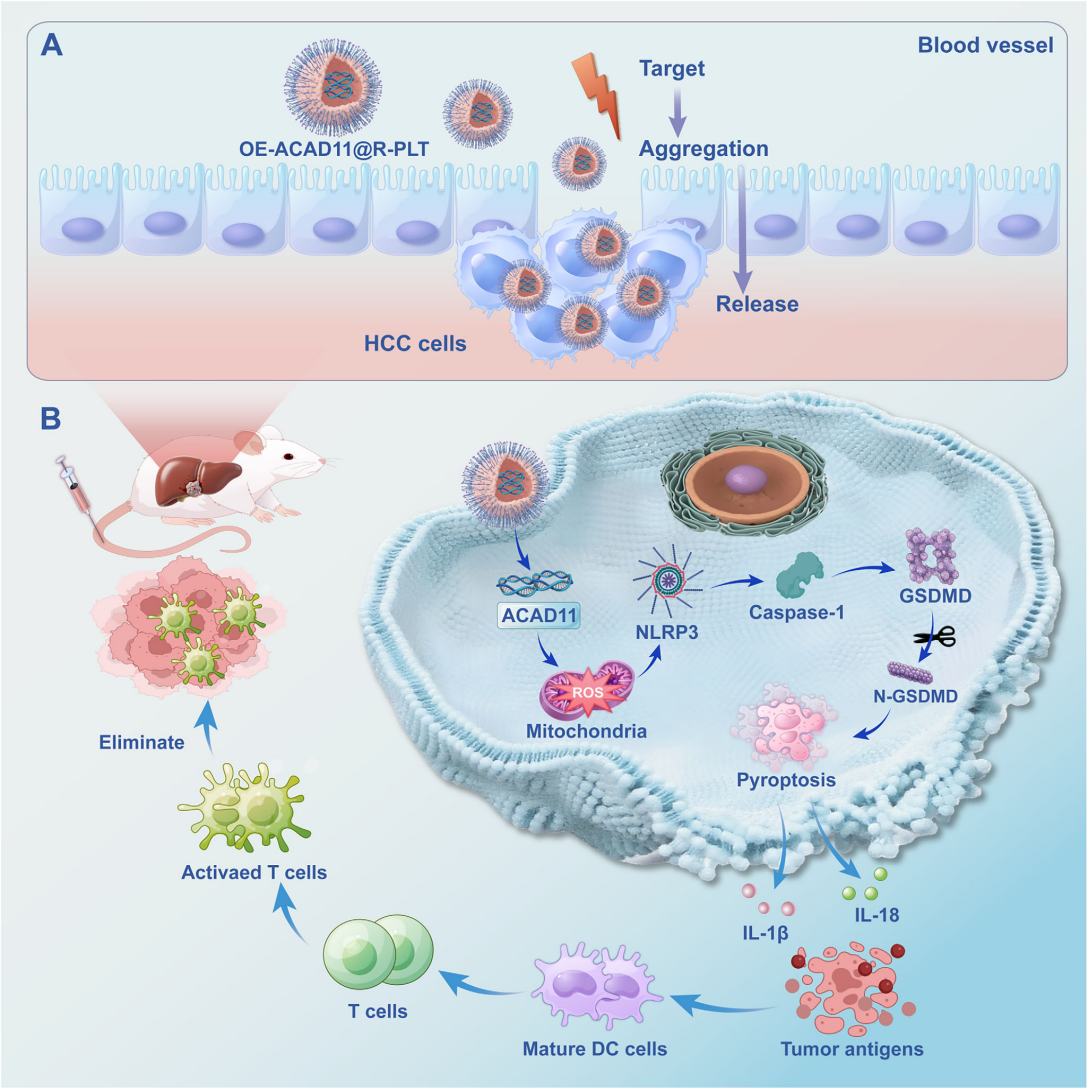
Schematic of the engineered platelet‐mediated delivery system (OE‐ACAD11@R‐PLT) as a targeted immunotherapy strategy for HCC. (A) Schematic of OE‐ACAD11@R‐PLT in targeted delivery and cargo release. OE‐ACAD11@R‐PLT targeted intravascular tumor sites, where it aggregated upon stimulation and released the therapeutic plasmid into HCC cells. (B) Illustration of the therapeutic mechanism of OE‐ACAD11@R‐PLT, which induces mitochondrial dysfunction to trigger the NLRP3/Caspase‐1/GSDMD‐mediated pyroptosis pathway. The induction of pyroptosis released tumor antigens and inflammatory cytokines (IL‐1β and IL‐18), which promoted DC maturation and activated T cells for tumor elimination.

## 2 Materials and Methods

### 2.1 Materials and Animals

Prostaglandin E1 (PGE1), Adenosine‐5'‐ Diphosphate (ADP) were purchased from Biobomei (Hefei, China). Cyclic RGDyk (cRGDyk) peptide, MPEG‐DSPE, and DSPE‐PEG‐MAL were supplied by Ponsure (Shanghai, China). These reagents were utilized for the synthesis and activation of OE‐ACAD11@R‐PLT.

Primary antibodies utilized for Western blot analysis: anti‐ACAD11 (ABclonal, A16153), anti‐NLRP3 (Selleck, F0335), anti‐Caspase‐1 (Selleck, F0461), anti‐GSDMD (BOSTE, M02842), anti‐CD41 (Proteintech, 24552‐1‐AP), anti‐CD61 (Proteintech, 18309‐1‐AP), anti‐CD62p (Selleck, F2555), anti‐Caspase‐3 (Selleck, F1080), anti‐GSDME (Selleck, F1701) and anti‐GAPDH (Abcam, ab8245). Anti‐mouse IgG (Proteintech, SA00001‐1) and anti‐rabbit IgG (Proteintech, RGAR001) were utilized as the HRP‐conjugated secondary antibodies.

Primary antibodies utilized for immunofluorescence (IF) and immunohistochemistry (IHC) staining: anti‐ACAD11 (Promab, PMB‐P07744), anti‐NLRP3 (Selleck, F0335), anti‐Caspase‐1 (Selleck, F0461), anti‐GSDMD (MCE, HY‐P86158) and anti‐CD62p (Selleck, F2555). Secondary antibody utilized for IF included Alexa Fluor 594‐conjugated goat anti‐rabbit IgG (Abcam, ab150080). HRP‐anti‐rabbit IgG (Proteintech, RGAR001) was employed as the secondary antibody for IHC.

Flow Cytometry‐related Reagents and Consumables: 70 um white cell filter mesh, 200 mesh (Falcon, 352350), ACK Lysis Buffer (Beyotime, C3702), anti‐CD45 (Ebioscience, 69‐0451‐82), anti‐CD3 (Ebioscience, 17‐0032‐80), anti‐CD4 (Ebioscience, 11‐0041‐81), anti‐CD8 (Ebioscience, 407‐0081‐80), anti‐CD25 (Biolegend, bl‐102033), anti‐CD11c (Ebioscience, 53‐0114‐80), anti‐MHC‐II (Ebioscience, 17‐5321‐81), anti‐CD11b (Ebioscience, 25‐0112‐81), anti‐CD86 (Biolegend, bl‐105031), Fixable Viability Dye eFluor780 (Ebioscience, 65‐0865‐14) and anti‐F4/80 (Biolegend, bl‐123133).

Sprague‐Dawley (SD) rats (female, 10 weeks old) and Institute of Cancer Research (ICR) mice (female, 6 weeks old,) were acquired from the Experimental Animal Center of Nantong University, China. The animals were placed in a temperature‐controlled (20°C–25°C) room with a relative humidity of 50%–70% and a 12/12 h light/dark cycle.

### 2.2 Clinical Sample

Fifty paired specimens of HCC and adjacent non‐tumorous tissues were harvested from patients at the Affiliated Hospital of Nantong University between May 2019 and May 2021. Each specimen was histologically confirmed by pathological examination before being snap‐frozen in liquid nitrogen and stored at −80°C for further investigation. None of the enrolled patients had undergone preoperative chemotherapy or radiotherapy. The protocol was reviewed and approved by the Ethics Committee of the Affiliated Hospital of Nantong University (approval no. 2024‐L004), with all participants providing written informed consent. Detailed clinicopathological profiles of the HCC cohort are summarized in Table S1.

### 2.3 Cell Culture

The human HCC cell lines HCC‐LM3, MHCC‐97H, and Huh7, the murine hepatoma cell line Hepatoma‐22, and the normal human hepatic cell line QSG‐7701 were supplied by the Cell Bank of the Chinese Academy of Sciences (Shanghai, China). HCC‐LM3, MHCC97H, and Huh7 cells were cultured in Dulbecco's modified Eagle's medium (DMEM; Corning, USA) supplemented with 10% fetal bovine serum, while QSG‐7701 cells and Hepatoma‐22 cells were maintained in RPMI‐1640 medium (Corning, USA). All cell lines were incubated at 37°C under 5% CO_2_ in a humidified atmosphere, with medium replenishment or subculturing performed every 2–3 days at split ratios of 1:2 to 1:4.

### 2.4 Synthesis of OE‐ACAD11@R‐PLT

Whole blood was collected from SD rats (female, 10 weeks old) via cardiac puncture using sodium citrate anticoagulant. PRP was isolated through density gradient centrifugation (800 g, 20 min, 22°C) with 1 µM PGE1 introduced to the collection tube to inhibit platelet activation during processing.

Then, 30 µg of OE‐ACAD11 was introduced into isolated platelet suspension with gentle pipetting, followed by ultrasonication at 100 W for 10 min at 25°C. The mixture was centrifuged at 1000 g for 10 min to remove supernatant, yielding OE‐ACAD11@PLT.

The OE‐ACAD11@PLT was then subjected to thiol modification, conjugated with cRGDyk pre‐functionalized using MPEG‐DSPE and DSPE‐PEG‐MAL via thiol‐maleimide reaction, and purified by centrifugation (1000 g, 10 min) to obtain OE‐ACAD11@R‐PLT.

### 2.5 Characterization of OE‐ACAD11@R‐PLT

Changes in the size distribution and surface potential profiles through the synthesis of OE‐ACAD11@R‐PLT were monitored via a Zetasizer Nano ZS90 (Malvern, UK). The morphology of PLTs and OE‐ACAD11@R‐PLT was visualized via SEM (JSM‐IT800, JEOL Co., Japan).

Confocal laser scanning microscopy (CLSM) was employed to verify the surface modification of cRGDyk on platelets. First, the cRGDyk peptide was labeled with CY5 (MCE, USA) via amino‐carboxyl coupling. The reaction was conducted in the dark for at least 30 min, followed by dialysis against phosphate‐buffered saline (PBS) for 12 h to eliminate unconjugated CY5. Simultaneously, platelets were labeled with FITC (Biobomei, China). Briefly, FITC was mixed with PLTs at a ratio of 500 µM per 10^5^ PLTs, and the mixture was incubated at 4°C with continuous stirring for 12 to 18 h. Unbound FITC was subsequently removed by dialysis to obtain FITC‐labeled PLTs. Finally, the CY5‐labeled cRGDyk was conjugated onto the surface of the FITC‐labeled PLTs. The resulting fluorescently labeled R‐PLTs were observed under a Leica TCS SP 8 STED 3X microscope (Wetzlar, Germany).

For the DNase protection assay, naked OE‐ACAD11 plasmid and intact or lysed OE‐ACAD11@R‐PLT were treated with DNase I, followed by plasmid‐specific qPCR analysis to evaluate plasmid protection within the platelet carrier. To evaluate the functional activity of the released plasmid, plasmids released from activated OE‐ACAD11@R‐PLT were collected and transfected into MHCC97H cells. ACAD11 mRNA expression was subsequently determined by qRT‐PCR and compared with that induced by freshly prepared OE‐ACAD11 plasmid.

### 2.6 In Vivo Tumor Model Generation and Drug Administration

ICR mice were procured from the Animal Center of Nantong University. Age‐matched (6 weeks) female mice were utilized throughout all experiments. To establish orthotopic HCC mouse models, anesthesia was induced in ICR mice (*n* = 9 per group) via an intraperitoneal injection of 1% pentobarbital sodium (50 mg/kg) prior to laparotomy and intraparenchymal injection of 1 × 10^6^ Hepatoma‐22 cells into the left hepatic lobe. On postoperative day 6, ICR mice were intravenously administered Cy5‐labeled OE‐ACAD11@PLT and Cy5‐labeled OE‐ACAD11@R‐PLT, respectively, via the tail vein. The total injection volume was 0.1 mL per animal. All animal experiments and procedures involving animal euthanasia were performed in accordance with the relevant institutional guidelines and were approved by the Animal Ethics Committee of Nantong University (approval no. P20240124‐010).

### 2.7 Targeted Behavior of OE‐ACAD11@R‐PLT

HCC cell line MHCC97H cells and human normal hepatocyte cell line QSG‐7701 were plated in the bottom chamber of the 24‐well plate and cultured to 70%–80% confluence. Subsequently, Transwell membranes with a pore size of 0.4 µM were placed in the wells, and OE‐ACAD11@R‐PLT was introduced to the upper chamber. After a 24 h co‐incubation, the membranes were retrieved. The monolayer cells in the bottom chamber were rinsed with PBS, and cell morphology was measured by SEM (JSM‐IT800, Japan Electron Optics Laboratory).

At 1 h post‐administration, mice were anesthetized (2% isoflurane v/v in O2) to observe the in vivo biodistribution of the formulation's fluorescence. Subsequently, the animals were euthanized via CO2 asphyxiation, and their hearts, livers, kidneys, and tumor tissues were excised. These harvested organs and tumors were then imaged to determine the ex vivo fluorescence distribution. Fluorescence image acquisition for both in vivo and ex vivo studies was carried out on an IVIS Lumina Series III platform (PerkinElmer, USA), followed by signal quantification using Living Image V4.4 software.

### 2.8 Tumor Penetration of OE‐ACAD11@R‐PLT

MHCC97H HCC cells were dissociated and plated in the ultra‐low attachment culture plates (Corning, USA) at a density of approximately 100 cells per well to facilitate spheroid formation. The culture medium was refreshed every 3 days, and the growth of spheroids was monitored microscopically. After approximately 10 days, the tumor spheroids were stained with Rhodamine. Subsequently, FITC‐labeled OE‐ACAD11@PLT or OE‐ACAD11@R‐PLT were added to the culture. After a 24 h co‐incubation period, the supernatant was carefully aspirated under microscopic guidance. The fluorescence distribution within the spheroids was measured by a Leica TCS SP8 STED 3X CLSM (Leica Microsystems, Wetzlar, Germany).

### 2.9 Quantitative Real‐Time PCR (qRT‐PCR)

Total RNA was isolated utilizing TRIzol reagent (Invitrogen, USA), reverse‐transcribed into cDNA with a commercial kit (Thermo Fisher Scientific, USA), and subsequently amplified via real‐time PCR employing Universal SYBR Green Fast qPCR Mix (ABclonal, China) on a LightCycler 480 instrument (Roche, Germany). The expression levels of ACAD11, HIGD1B, PHYHD1, and TTC9 were quantified by a comparative threshold cycle (2^−ΔΔCt^) protocol, with GAPDH as the endogenous reference control. The detailed primer sequences are listed in Table S2. All primers were purchased from Sangon Biotech (Shanghai, China).

### 2.10 Western Blot Analysis

Total protein was isolated from cells and platelets following established procedures. After quantifying protein concentrations, aliquots were denatured at 97°C for 5–10 min in a water bath. Denatured samples were resolved by SDS‐PAGE (Beyotime, China) and electrotransferred onto methanol‐activated PVDF membranes (Millipore, USA). Membranes were blocked with 5% skim milk in TBST for 120 min at room temperature, then incubated with primary antibodies overnight at 4°C. Following TBST washes, membranes were probed with horseradish peroxidase‐conjugated secondary antibodies for 60 min at room temperature. Protein signals were ultimately determined by a Tanon‐5200 automated chemiluminescence imaging system (Tanon, China).

### 2.11 Cell Transfection

HCC cells were transfected with a variety of constructs, including pLenti‐CMV negative control (vector) and pLenti‐CMV‐ACAD11 (OE‐ACAD11), as well as si‐ACAD11 (si‐ACAD11‐1, si‐ACAD11‐2, and si‐ACAD11‐3) and siRNA negative control (si‐NC). These reagents were supplied by Obio Technology (Shanghai, China). Transfection was performed using the Lipofectamine 3000 (Invitrogen, USA) at a cell density of ∼70%. After a 48‐h incubation, transfection efficiency was verified via RT‐qPCR. Details regarding the siRNA vectors are listed in Tables S3 and S4.

### 2.12 Cell Proliferation

Cell proliferation was analyzed through parallel methodologies. For metabolic activity assessment, HCC cells were plated into 96‐well plates at 3 × 10^3^ cells/well and probed with 10 µL CCK‐8 reagent (MedChemExpress, China) at 24, 48, and 72‐h intervals; post 2 h incubation at 37°C, absorbance was determined at 450 nm via a microplate reader (Thermo Fisher, USA). Concurrently, DNA synthesis was evaluated by pulsing cells in 48‐well plates with 100 µL EdU working solution (Beyotime, China) for 2 h, succeeded by fixation in 4% paraformaldehyde (15 min), permeabilization with 0.3% Triton X‐100 (15 min), blocking in 3% BSA/PBS, nuclear staining with Hoechst 33258 (1 µg/mL, 15 min), and fluorescence imaging using an Olympus IX73 microscope (Japan).

### 2.13 Clone Formation Assay

Cell growth was assessed by seeding single‐cell suspensions in 6‐well plates at 1 × 10^4^ cells/well (triplicate wells), with complete medium replacement every 3 days. Cultures were terminated for crystal violet staining when control groups reached 100% confluency. For clonogenic potential evaluation, cells were plated in 12‐well plates at densities ranging from 1 × 10^2^ to 1 × 10^3^ cells/well (triplicate), maintained for 12–21 days with medium changes every 3 days, and processed at control group confluency.

### 2.14 Wound‐Healing Assay

Six‐well plates were pre‐marked with reference lines on the underside. Cells were seeded to achieve 100% confluent monolayers overnight. Uniform wounds were created across cell monolayers using a sterile 200 µL pipette tip held perpendicular to the plate surface. After removing dislodged cells with three PBS washes and aspirating residual liquid, serum‐free medium was added. The culture plates were maintained at 37°C in a 5% CO_2_ incubator. Subsequently, the wound gaps were photographed at 0 and 24 h via a phase‐contrast microscope. Migration rates were quantified by measuring wound closure percentage in ImageJ (NIH, USA).

### 2.15 Cell Invasion and Migration Assay

Invasiveness was assessed by 8‐µm pore Transwell chambers pre‐coated with 100 µl diluted Matrigel (Corning, USA). HCC cells suspended in serum‐free medium were plated into the upper chamber, while the basolateral compartment contained complete medium supplemented with FBS. After 48 h incubation, membranes were preserved in 4% paraformaldehyde and stained with 0.5% crystal violet. Migratory potential was evaluated in parallel using identical chambers and procedures without Matrigel coating. For both assays, non‐invading/migrating cells were removed by rinsing, and traversed cells were quantified by microscopic counting.

### 2.16 Cellular ROS Detection

Intracellular ROS generation was measured by a ROS Assay Kit (Beyotime, S0033) according to the instructions of manufacturer. Treated cells were probed with DCFH‐DA 10 µM for 20 min at 37°C. After removing the excess dye via washing, ROS‐mediated DCF fluorescence was imaged using a Zeiss microscope system (Zeiss, Oberkochen, Germany).

### 2.17 Mitochondrial Membrane Potential Assay

Mitochondrial membrane potential was assessed by a JC‐1 Assay Kit (Beyotime, C2006) according to the instructions of manufacturer. Briefly, the treated MHCC97H cells were stained with JC‐1 working solution for 20 min at 37°C protected from light. The cells were then washed twice with ice‐cold buffer and immersed in fresh culture medium. The signals were visualized by a microscope system (Zeiss, Oberkochen, Germany).

### 2.18 Immunofluorescence (IF)

Cell fixation and permeabilization were performed using 4% PFA (15 min) and Triton X‐100 (20 min), respectively. To minimize non‐specific binding, samples were blocked with 5% bovine serum albumin (BSA) at 37°C for 1 h. Following overnight incubation with primary antibodies at 4°C, cells were incubated with secondary antibodies for 1 h at 37°C. For nuclear visualization, the samples were incubated with DAPI for 3 min, and images were acquired via fluorescence microscopy.

### 2.19 Enzyme‐Linked Immunosorbent Assay (ELISA)

The intratumoral concentrations of HMGB1, IL‐1β, IL‐18, and LDH were quantified using ELISA kits (Jianglai Biology, Shanghai, China) following the instructions of manufacturer. In brief, tumor tissues were weighed and subsequently disrupted in chilled PBS. Centrifugation of the homogenates was performed at 5000 rpm for 10–20 min at 4°C to obtain the supernatants for analysis. The optical density (OD) values were measured at 450 nm by microplate reader.

### 2.20 Flow Cytometry Assay

Single‐cell suspensions were prepared according to standardized protocols and adjusted to 1 × 106 cells/100 µl using an automated cell counter. Cells were incubated with fluorophore‐conjugated antibodies per manufacturer's specifications (4°C, dark, 30 min), washed twice with ice‐cold PBS, resuspended in 300 µL staining buffer, and immediately analyzed on an Attune NxT Acoustic Focusing Cytometer (Thermo Fisher, USA). Fluorescence‐positive populations were quantified across multiple channels using Attune NxT Software v3.1.

### 2.21 Immunohistochemistry (IHC)

Following standard deparaffinization and rehydration, tissue sections underwent heat‐mediated antigen retrieval using citrate buffer (pH 6.0). Endogenous peroxidase was quenched using 3% H_2_O_2_ for 15 min. Sections were then blocked with 3% BSA for 30 min at room temperature before being stained with primary antibodies overnight at 4°C, subsequent to exposure to horseradish peroxidase‐conjugated secondary antibody for 50 min at room temperature. Chromogenic detection proceeded with 3,3'‐diaminobenzidine substrates under microscopic monitoring, followed by hematoxylin counterstaining to visualize nuclei. Ultimately, the sections were subjected to dehydration in ethanol, cleared in xylene, mounted with resin, and examined by microscopy.

### 2.22 Hematoxylin and Eosin (H&E) Staining

For the evaluation of systemic safety of OE‐ACAD11@R‐PLT, major organs including the heart, spleen, lungs, and kidneys were harvested from the treated mice. The tissues were fixed in paraformaldehyde and subsequently embedded in paraffin. Serial sections with a thickness of 3 µm were prepared for routine histological assessment and stained with H&E. Ultimately, representative images were acquired via an optical microscope (IX71, Olympus, Tokyo, Japan).

### 2.23 Statistical Analysis

Statistical analyses were performed using SPSS 20.0. Data are presented as mean ± standard deviation (SD). Two‐tailed Student's t‐tests were used for pairwise comparisons. One‐way ANOVA was used for multiple‐group comparisons where applicable, and two‐way ANOVA was used for time‐course or longitudinal data. Survival differences were analyzed using the log‐rank test. Sample sizes (n) are indicated in the corresponding figure legends. A two‐sided *P* < 0.05 was considered statistically significant.

## 3 Results

### 3.1 ACAD11 Is Downregulated in HCC and Inhibits Tumor Progression

Initially, we identified four genes (ACAD11, HIGD1B, PHYHD1, and TTC9) that were most significantly dysregulated using datasets retrieved from the Gene Expression Omnibus (GEO). Further refinement was performed via volcano plot analysis (Figure 1A). Furthermore, it was confirmed by qRT‐PCR analysis that ACAD11 expression was markedly reduced in HCC tissues compared to adjacent normal tissues (*P* < 0.001). Conversely, no discernible discrepancies were observed in the expression levels of HIGD1B, PHYHD1, and TTC9 between the two tissue types (Figure 1B). Moreover, Kaplan‐Meier survival analysis was conducted for these four dysregulated genes based on The Cancer Genome Atlas (TCGA) database, and it was revealed that ACAD11 exhibited the most significant correlation with survival rates in HCC patients (Figure S1). ACAD11 protein expression was demonstrated by Western blot analysis to be significantly decreased in HCC tissues compared to adjacent normal tissues (*P* < 0.0001) (Figure 1C). Consistent with this, markedly decreased ACAD11 expression levels were revealed by IHC in HCC tissues compared to adjacent normal tissues (Figure S2). Consequently, ACAD11 was selected for further investigation. Subsequently, it was indicated by correlation analysis of clinicopathological characteristics that tumor size, tumor differentiation, and tumor stage were all significantly associated with decreased ACAD11 expression in HCC patients (Figure 1D–F and Table S1). To evaluate its prognostic significance, Kaplan‐Meier (KM) survival curves were generated to evaluate the survival probabilities in our institutional HCC dataset. (Figure 1G). Low ACAD11 expression was significantly associated with reduced median survival times in patients (*P* = 0.0012). Meanwhile, the diagnostic value of ACAD11 was estimated by Receiver Operating Characteristic (ROC) curve analysis, and an Area Under the Curve (AUC) of 0.834 was yielded for HCC (Figure 1H). These findings collectively indicate that ACAD11 downregulation may play a pivotal role in HCC.

**FIGURE 1:**
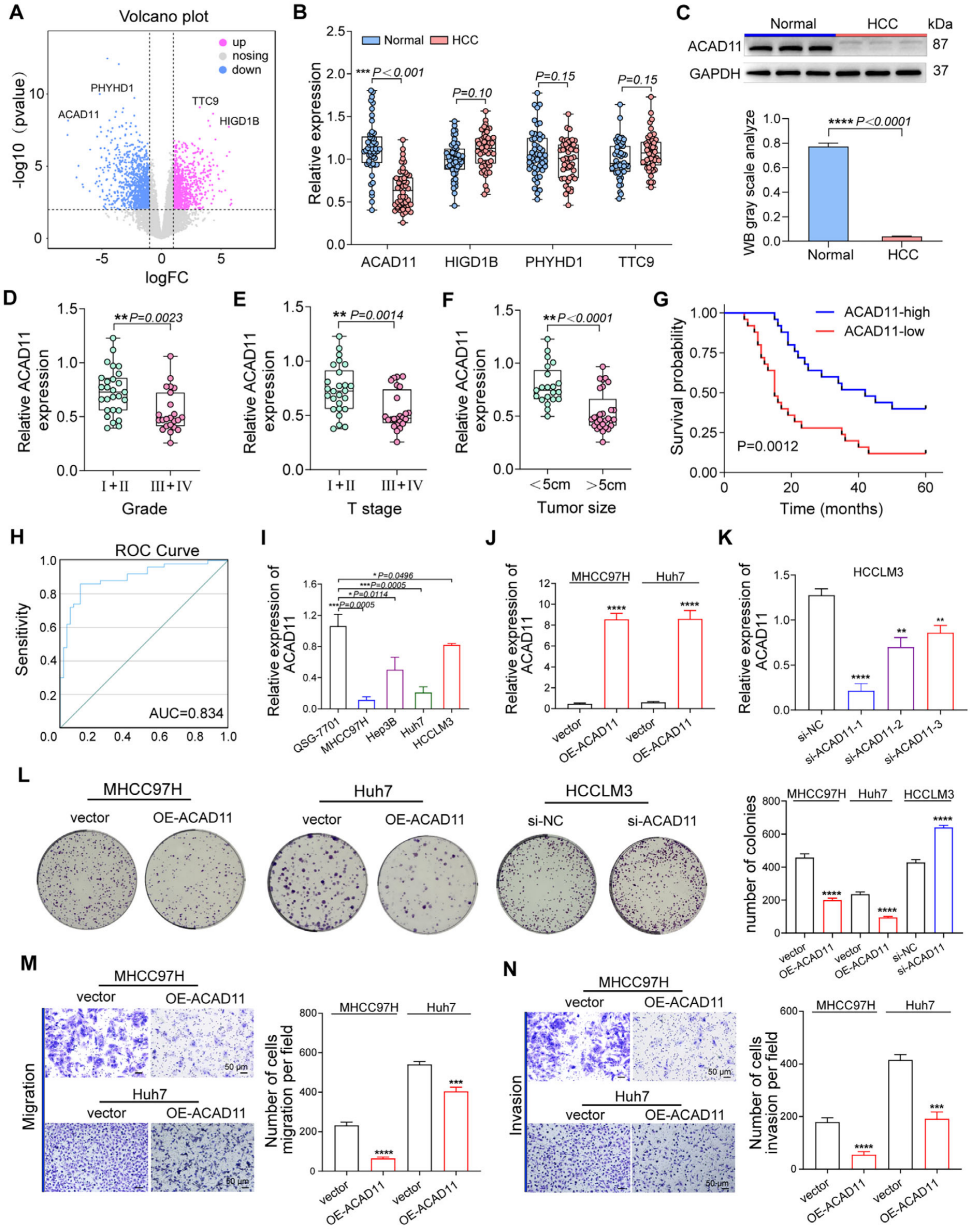
ACAD11 is downregulated in HCC and inhibits tumor progression. (A) Volcano plot visualizing the distribution of deregulated genes in HCC derived from the GEO database. (B) The mRNA expression levels of ACAD11, HIGD1B, PHYHD1, and TTC9 in 50 paired HCC tissues and adjacent non‐tumor tissues. (C) Western blot analysis of ACAD11 expression in HCC and adjacent non‐tumor tissues. Lower panel: Semi‐quantitative densitometric analysis. (D‐F) Correlation analysis between ACAD11 expression and clinicopathological characteristics, including pathological grade (D), T stage (E), and tumor size (F). (G) KM curves of HCC patients stratified by ACAD11 expression. (H) ROC curve analysis of ACAD11 for the differential diagnosis of HCC and normal tissues. (I) ACAD11 expression in the normal liver cell line and HCC cell lines. (J, K) Transfection efficiency of OE‐ACAD11 (J) and si‐ACAD11 (K) in HCC cells. (L) Colony formation assay of HCC cells after transfection with OE‐ACAD11 or si‐ACAD11. (M, N) Transwell assays evaluating the migration (M) and invasion (N) capabilities of HCC cells treated with OE‐ACAD11. Cell‐based quantitative data are presented as mean ± SD (*n* = 3). Pairwise comparisons were performed using two‐tailed Student's t‐tests. Survival differences were analyzed using the log‐rank test. * P < 0.05, ** P < 0.01, *** P < 0.001 and **** P < 0.0001.

In order to explore the biological function of ACAD11, its expression profile in HCC cell lines was first examined. It was revealed by RT‐qPCR analysis that ACAD11 was significantly downregulated in all four tested HCC cell lines in comparison with the normal hepatocyte line QSG‐7701 (Figure 1I). For functional perturbation studies, we constructed the pLenti‐CMV‐ACAD11 (OE‐ACAD11) overexpression vector for ACAD11 upregulation and si‐ACAD11‐1/3 siRNA‐based RNA interference vectors for targeted ACAD11 knockdown, respectively (Tables S3 and S4). Transfection efficiency was validated, with results indicating significantly elevated ACAD11 expression levels in MHCC97H and Huh7 cells transfected with OE‐ACAD11 compared to the vector control group (Figure 1J). Meanwhile, ACAD11 expression was effectively inhibited by both si‐ACAD11‐1 and si‐ACAD11‐3 in LM3 cells. Following rigorous quantitative assessment via RT‐qPCR, si‐ACAD11‐1, which exhibited the optimal downregulation, was chosen for subsequent experiments and designated as si‐ACAD11. A negative control siRNA (si‐NC) served as the control (Figure 1K).

Functional validation via CCK‐8 and soft agar assays revealed that upregulating ACAD11 markedly suppressed the proliferative and clonogenic potential of MHCC97H and Huh7 cells relative to vector controls. Conversely, LM3 cells exhibited markedly enhanced proliferation and colony formation capabilities following ACAD11 knockdown relative to the si‐NC control group (Figure S3 and Figure 1L). In addition, wound healing and Transwell assays confirmed that OE‐ACAD11 treatment markedly diminished cancer cell migration and invasion capabilities (Figure S4 and Figure 1M,N), whereas si‐ACAD11 treatment significantly enhanced LM3 cell migration and invasion capabilities (Figure S5).

In summary, our findings establish ACAD11 as a critical tumor suppressor in HCC, acting through the dual suppression of cell proliferation and metastatic dissemination. However, the specific molecular mechanisms underlying its anti‐tumor efficacy remains to be fully elucidated. This prompts us to further investigate the downstream signaling pathways modulated by ACAD11.

### 3.2 ACAD11 Overexpression Activates NLRP3/Caspase‐1/GSDMD‐Mediated Pyroptosis and Enhances Therapeutic Efficacy

Time‐course morphological observation revealed progressive pyroptosis‐associated changes following OE‐ACAD11 transfection, with characteristic cell swelling and balloon‐like membrane protrusions becoming evident at 12 h and more pronounced at 24 h (Figure S6). These time‐dependent morphological changes supported the progressive induction of pyroptosis following OE‐ACAD11 overexpression. Consistently, analysis of the culture supernatants revealed significantly increased levels of LDH, IL‐1β, and IL‐18 in OE‐ACAD11‐transfected cells compared with vector‐control cells (Figure S7), further supporting the induction of pyroptosis.

To explore the signaling pathways involved, RNA sequencing was performed on OE‐ACAD11 versus vector‐control MHCC97H cells. A total of 1699 differentially expressed genes (DEGs) were identified (944 upregulated, 755 downregulated), indicating systemic biological reprogramming (Figure 2B,C). It was revealed by GO analysis that the regulation of cellular response to stimulus was significantly altered (Figure S8). It is noteworthy that through the Kyoto Encyclopedia of Genes (KEGG) pathway enrichment analysis, the NOD‐like receptor signaling pathway was identified as a core regulatory node (Figure 2D). Heatmap profiling of the NOD‐like signaling pathway confirmed the simultaneous elevation of NLRP3, Caspase‐1, and GSDMD, suggesting that ACAD11 drives pyroptosis via this axis (Figure 2E). Western blot analysis further supported the transcriptomic findings, showing increased expression of NLRP3, CASP‐1, and GSDMD following ACAD11 overexpression (Figure 2F). In addition, increased cleaved CASP‐1 and GSDMD‐N were observed, further supporting activation of the CASP‐1/GSDMD pyroptotic pathway (Figure S12).

**FIGURE 2:**
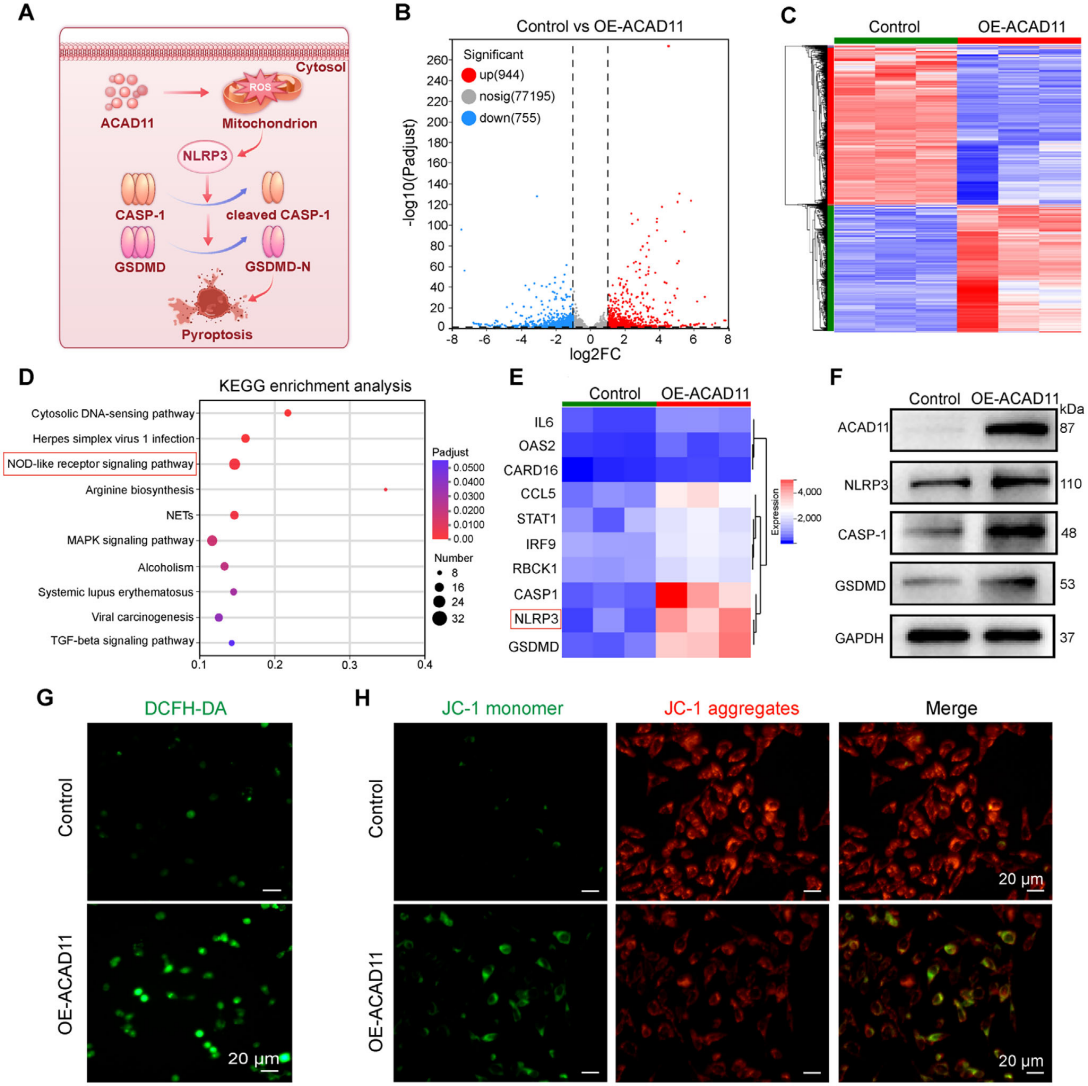
Upregulation of ACAD11 activates pyroptosis mediated by NLRP3/Caspase‐1/GSDMD. (A) Schematic illustration of ACAD11‐induced pyroptosis via the mitochondrial ROS‐NLRP3 axis. (B) Volcano plot analysis of DEGs between the Control and OE‐ACAD11 groups. (C) Hierarchical clustering heatmap of DEGs between the Control and OE‐ACAD11 groups. (D) KEGG pathway enrichment illustration highlighting the top 10 significantly enriched pathways between the Control and OE‐ACAD11 groups. The NOD‐like receptor signaling pathway is highlighted. (E) Heatmap illustrating the expression profiles of genes enriched in the NOD‐like receptor signaling pathway. (F) Western blot analysis of ACAD11, NLRP3, Caspase‐1, and GSDMD. (G) Intracellular ROS levels determined by DCFH‐DA staining. (H) Mitochondrial membrane potential determined by JC‐1 staining.

ACAD11 is widely verified as a mitochondrial‐localized fatty acid oxidation enzyme [28]. Mitochondrial dysfunction, characterized by ROS accumulation and loss of membrane potential, is recognized as a key upstream signal for NLRP3 inflammasome activation [29]. Based on the transcriptomic data, it was hypothesized that ACAD11 overexpression triggered the ROS‐mediated NLRP3/Caspase‐1/GSDMD pyroptosis signaling pathway by inducing mitochondrial stress (Figure 2A). DCFH‐DA staining revealed a marked elevation of intracellular ROS levels in the OE‐ACAD11 group, confirming mitochondrial perturbation (Figure 2G, Figure S9). Concurrently, JC‐1 staining revealed a marked drop in the red/green ratio, indicating a collapse of mitochondrial membrane potential (Figure 2H, Figure S10). To further determine whether ROS accumulation mediates the activation of pyroptotic signaling following ACAD11 overexpression, NAC was used as a ROS scavenger. NAC treatment markedly attenuated the increase in intracellular ROS induced by ACAD11 overexpression, with ROS levels returning close to those observed in vector‐control cells (Figure S11). Consistently, Western blot analysis showed that NAC treatment attenuated the activation of the NLRP3/Caspase‐1/GSDMD pathway induced by ACAD11 overexpression (Figure S12). These findings suggest that ROS acts as an intermediate signal linking ACAD11‐induced mitochondrial stress to downstream pyroptotic signaling, rather than supporting a direct regulatory effect of ACAD11 on the pyroptosis machinery.

Given that ROS may also promote pyroptosis through the CASP‐3/GSDME pathway, we further examined CASP‐3 and GSDME expression and activation following ACAD11 overexpression. Western blot analysis showed no evident increase in cleaved CASP‐3 or GSDME‐N, suggesting that the CASP‐3/GSDME pathway was not prominently activated under these conditions (Figure S13). These findings further support the predominant involvement of the NLRP3/Caspase‐1/GSDMD pathway in ACAD11‐induced pyroptosis.

However, preliminary in vivo studies revealed that traditional viral delivery was inefficient. Immunostaining showed no significant ACAD11 upregulation in treated orthotopic HCC tumors (Figure S14). This delivery barrier underscores the critical need for an optimized vector system to realize the therapeutic potential of ACAD11.

### 3.3 Construction of OE‐ACAD11@R‐PLT

To circumvent the delivery limitations of traditional viral vectors, a targeted delivery system named OE‐ACAD11@R‐PLT was designed. The targeting peptide cRGDyk was conjugated to the platelet surface, followed by OE‐ACAD11 encapsulation to yield the final system (Figure 3A). Loading of the plasmid cargo resulted in a minor expansion in the size of OE‐ACAD11@R‐PLT relative to naive platelets (Figure 3B). The zeta potential of OE‐ACAD11@R‐PLT was observed to be comparable to native platelets, indicating the successful encapsulation of OE‐ACAD11 (Figure 3C). To further confirm the internal encapsulation of OE‐ACAD11, a DNase protection assay combined with plasmid‐specific qPCR was performed. DNase I markedly degraded naked plasmid DNA, whereas the plasmid signal was largely preserved in intact OE‐ACAD11@R‐PLTs but significantly decreased after platelet lysis, indicating that OE‐ACAD11 was predominantly encapsulated within the platelets rather than surface‐adsorbed (Figure S15). We next evaluated whether the plasmid released from activated platelets retained its functional activity. The released plasmid was collected and subsequently transfected into MHCC97H cells, followed by qRT‐PCR analysis of ACAD11 expression. The released OE‐ACAD11 plasmid markedly increased ACAD11 mRNA expression compared with the released Vector plasmid, while its transfection activity was comparable to that of freshly prepared OE‐ACAD11 plasmid. These results demonstrate that the OE‐ACAD11 plasmid retained its functional integrity after platelet loading and subsequent release (Figure S16).

**FIGURE 3:**
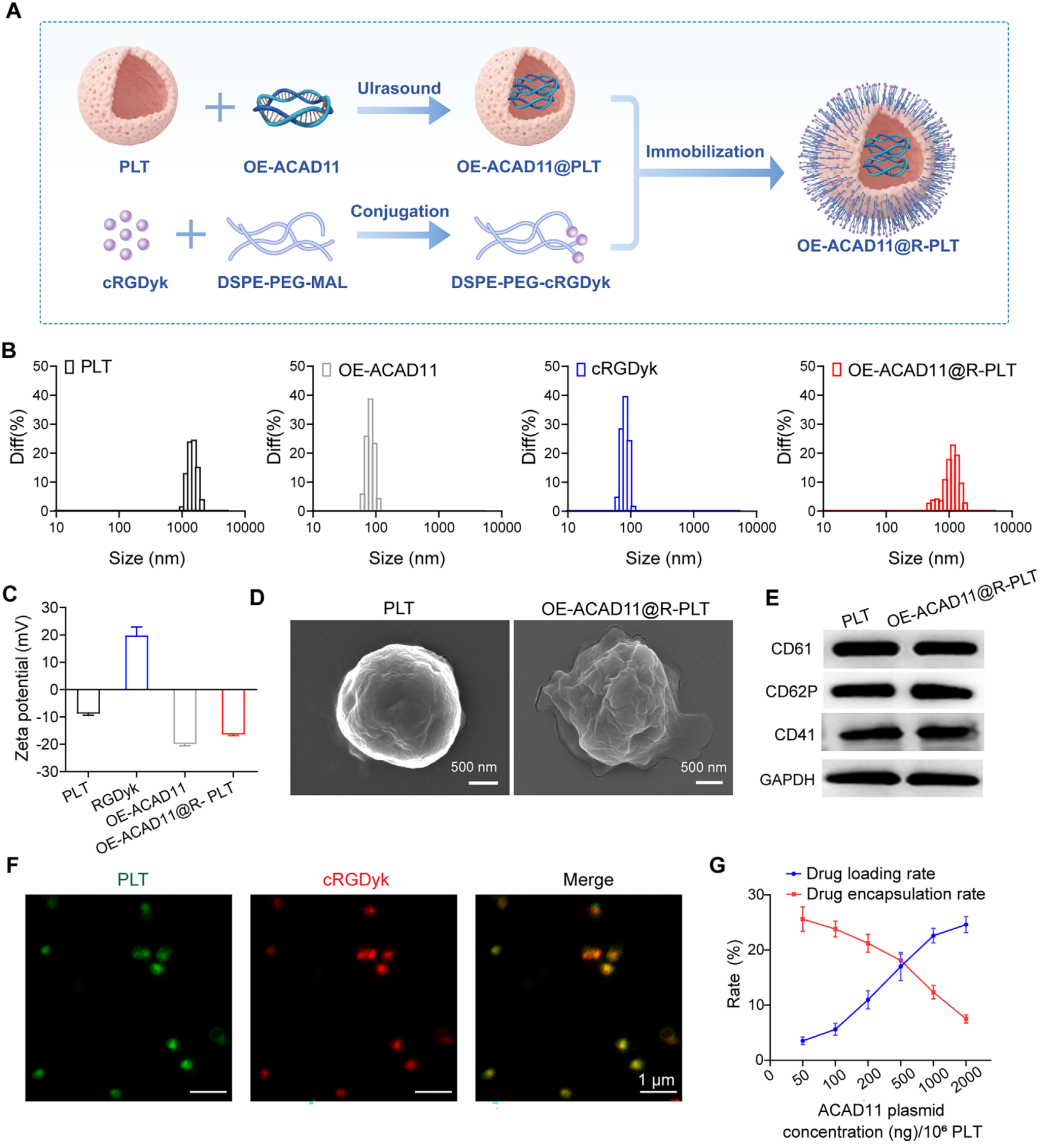
The construction and characterization of OE‐ACAD11@R‐PLT. (A) Schematic representation of the construction of OE‐ACAD11@R‐PLT. (B) The size distribution of PLT, OE‐ACAD11, cRGDyk, and OE‐ACAD11@R‐PLT. (C) The zeta potential of PLT, cRGDyk, OE‐ACAD11, and OE‐ACAD11@R‐PLT. (D) SEM images of raw PLT and OE‐ACAD11@R‐PLT. (E) The marker protein (CD62P, CD61, CD41, and GAPDH) in raw PLT and OE‐ACAD11@R‐PLT. (F) CLSM results of OE‐ACAD11@R‐PLT. Green, FITC‐labeled PLTs. Red, Cy5‐labeled cRGDyk. (G) Drug loading efficiency and encapsulation rate of OE‐ACAD11@R‐PLT.

As observed by SEM, an inactivated spherical morphology with increased roughness was maintained by OE‐ACAD11@R‐PLT, indicating preserved structural integrity (Figure 3D). This inert state was preserved by PGE1, a potent inhibitor of platelet activation and aggregation [30]. As shown in Figure S17, OE‐ACAD11@R‐PLT exhibited favorable stability in vitro, maintaining steady levels for 6 days before a marked decline. Moreover, Figure 3E demonstrated that OE‐ACAD11@R‐PLT retained key functional proteins (CD41, CD61, CD62P) at levels consistent with native platelets, verifying the preservation of membrane integrity. Furthermore, the OE‐ACAD11@R‐PLT complex was characterized using CLSM. Dual fluorescence signals were observed: green fluorescence emitted by FITC‐labeled PLTs and red fluorescence emitted by Cy5‐labeled cRGDyk (Figure 3F). The merged image displayed significant colocalization (appearing yellow) of the cRGDyk‐derived red fluorescence and the PLTs‐associated green fluorescence, confirming the successful surface modification with cRGDyk. To further semiquantitatively evaluate the surface modification of cRGDyk, flow cytometry was performed using fluorescently labeled cRGDyk. R‐PLTs exhibited a marked rightward shift in fluorescence intensity compared with unmodified PLTs, accompanied by a significantly increased median fluorescence intensity (MFI). Moreover, comparable MFI values were observed among three independently prepared batches, indicating consistent cRGDyk surface functionalization and good batch‐to‐batch reproducibility (Figure S18).

As platelets lack a cell nucleus, there is ample space available for drug loading [31]. Therefore, the drug loading capacity of OE‐ACAD11@R‐PLT was investigated. As shown in Figure 3G, an increase in OE‐ACAD11 plasmid concentration led to superior drug loading capacity, whereas the encapsulation efficiency exhibited an inverse trend. Balancing cost‐effectiveness with design requirements, an OE‐ACAD11 plasmid concentration of 500 ng/10^6^ PLTs was chosen for following experiments.

### 3.4 Targeted Behavior of OE‐ACAD11@R‐PLT

Platelets possess innate tumor‐homing capabilities and have been utilized as tumor drug carriers in previous studies [32]. However, in the clinical context, HCC is often accompanied by an inflammatory microenvironment in the liver. A critical limitation of using native platelets as carriers is their intrinsic tendency to bind to inflammatory markers on the surface of inflamed endothelial cells [33]. This often leads to substantial sequestration of platelets in non‐tumor liver parenchyma, resulting in a significant off‐target effect that restricts their effective enrichment within the tumor. The cRGDyk peptide is a cyclic pentapeptide containing the classic RGD motif, which specifically binds to integrin α_v_β_3_ [26, 34]. Integrin α_v_β_3_ is highly upregulated on the surface of malignant cells while remaining undetectable on healthy hepatocytes [35]. To mitigate non‐specific platelet aggregation, platelet surfaces were modified with cRGDyk peptides, enabling them to actively target the integrin α_v_β_3_ overexpressed at liver tumor sites. To validate the in vivo efficacy of this strategy, orthotopic HCC mouse models were injected with Cy5‐labeled native platelets (OE‐ACAD11@PLT) and cRGDyk‐modified platelets (OE‐ACAD11@R‐PLT), respectively. Both formulations were loaded with the OE‐ACAD11 plasmid to exclude the influence of the loading process. In vivo fluorescence imaging revealed distinct distribution patterns between OE‐ACAD11@PLT and OE‐ACAD11@R‐PLT. A relatively weak fluorescence signal was observed in the hepatic region of the OE‐ACAD11@PLT group at 2 h post‐injection, whereas OE‐ACAD11@R‐PLT produced a strong hepatic‐region signal as early as 0.5 h after administration. More importantly, ex vivo fluorescence imaging enabled direct comparison between tumor and non‐tumorous liver regions. OE‐ACAD11@PLT exhibited substantial and diffuse fluorescence in the non‐tumorous liver tissue, whereas OE‐ACAD11@R‐PLT showed preferential enrichment at the tumor site accompanied by markedly reduced fluorescence in the surrounding non‐tumorous liver tissue (Figure 4A). To further clarify the anatomical localization of the tumor regions used for fluorescence analysis, gross images of the corresponding excised tumor‐bearing livers were additionally provided (Figure S19). Quantitative analysis further confirmed that OE‐ACAD11@PLT was predominantly distributed in the liver and kidneys, whereas OE‐ACAD11@R‐PLT exhibited significantly enhanced tumor accumulation together with reduced distribution in the liver and kidneys. These reciprocal changes in tumor and hepatic distribution indicate that cRGDyk modification improves tumor‐to‐liver selectivity rather than merely increasing overall platelet accumulation (Figure S20, Figure 4B). Furthermore, organs were collected 24 h post‐administration for imaging. It was found that the OE‐ACAD11@R‐PLT group exhibited significant enrichment within the tumor, and the total fluorescence intensity was markedly stronger than that of the OE‐ACAD11@PLT group. These results suggested that R‐PLT significantly prolonged in vivo retention time by enhancing specific targeting capabilities (Figure S21).

**FIGURE 4:**
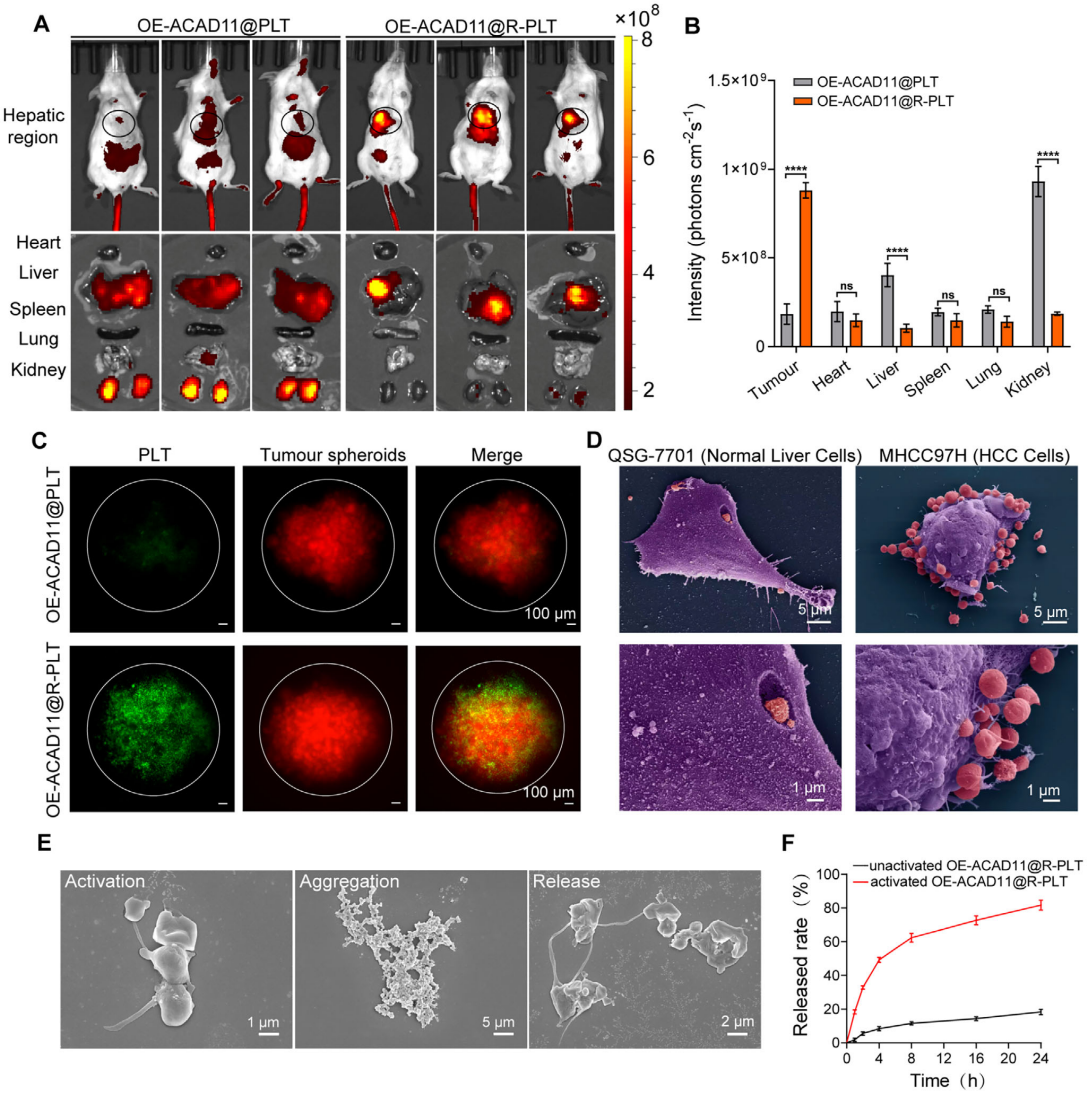
Targeted behavior of OE‐ACAD11@R‐PLT. (A) In vivo fluorescence imaging of orthotopic HCC‐bearing mice and ex vivo fluorescence imaging of major organs. Tumor and non‐tumorous liver regions in the excised livers were separately indicated for fluorescence analysis. (B) Quantification of fluorescence intensity in tumors, non‐tumorous liver tissues, and major organs. (C) CLSM images showing the penetration of OE‐ACAD11@PLT and OE‐ACAD11@R‐PLT into 3D tumor spheroids. (D) Pseudo‐colored SEM images of OE‐ACAD11@R‐PLT adhesion to QSG‐7701 and MHCC97H cells. (E) SEM images showing the activation, aggregation, and release process of OE‐ACAD11@R‐PLT. (F) The drug release profiles of OE‐ACAD11@R‐PLT triggered by activation. Data are presented as mean ± SD (*n* = 3). Statistical significance was assessed using a two‐tailed Student's t‐test. **** P < 0.0001. ns, nonsignificant (*P* > 0.05).

3D tumor spheroid technology can remodel the tumor microenvironment in vitro, accurately mimicking inherent properties of solid tumors. Herein, 3D tumor spheroids were employed to assess the impact of cRGDyk peptide addition on tumor targeting and penetration capabilities (Figure 4C). Rhodamine‐labeled 3D spheroids were incubated with FITC‐labeled OE‐ACAD11@PLT or OE‐ACAD11@R‐PLT. CLSM imaging revealed that while the unmodified PLT group showed minimal internal fluorescence, OE‐ACAD11@R‐PLT accumulated significantly within the spheroid core. These results confirmed that cRGDyk modification effectively enhanced tumor targeting and deep penetration capabilities. Then, to intuitively demonstrate the specific targeting ability of OE‐ACAD11@R‐PLT toward HCC cells, MHCC97H cells (HCC cells) or QSG‐7701 cells (human normal hepatocytes) were placed in the lower chamber of a Transwell apparatus, while OE‐ACAD11@R‐PLT was seeded into the upper compartment. After 24 h of incubation, OE‐ACAD11@R‐PLT migrated to the lower chamber through the 0.4 µm pore size membrane (Figure S22). SEM was employed to evaluate the binding of OE‐ACAD11@R‐PLT to cells, and the images were pseudo‐colored (Cells: purple; OE‐ACAD11@R‐PLT: red). As shown in Figure 4D, OE‐ACAD11@R‐PLT adhered extensively to MHCC97H cells, whereas almost no adhesion was observed on QSG‐7701 cells. Collectively, these results demonstrate that the addition of the cRGDyk peptide reduces the interference of the inflammatory hepatic background on platelets and enhances their targeting and penetration capabilities toward tumor cells.

Upon activation by tumor‐secreted factors (e.g., ADP), platelets are migrated to the tumor site and stimulated to secrete bioactive molecules [36]. Platelets are efficiently activated and their morphology and granule release are altered by ADP, which is spontaneously secreted by malignant tumor cells. This plasticity is exploited for targeted therapeutic delivery [37]. To validate the drug release of OE‐ACAD11@R‐PLT upon tumor activation, ADP was employed to simulate the HCC environment and observe the in vitro activation of OE‐ACAD11@R‐PLT. CD62P is a crucial component for PLT recognition and adhesion to tumor cells [38]. As shown in the IF images, CD62P expression levels were significantly elevated following ADP activation (Figure S23). SEM results revealed that following incubation with ADP, OE‐ACAD11@R‐PLT underwent a morphological transition from spherical to spindle‐shaped, extending multiple filamentous pseudopodia. This was followed by aggregation, culminating in the release of the plasmid drug OE‐ACAD11 (Figure 4E). Then, the size distributions of the activated OE‐ACAD11@R‐PLT and the released drug were determined. The results showed peaks at 82 and 1152 nm, reflecting the rapid release of the drug from within the OE‐ACAD11@R‐PLT upon activation (Figure S24). Furthermore, the drug release rate of OE‐ACAD11@R‐PLT during the activation process was examined. As depicted in Figure 4F, the activated OE‐ACAD11@R‐PLT exhibited a rapid release profile, with the predominant fraction of the encapsulated payload being liberated within the 24 h timeframe. Conversely, unactivated OE‐ACAD11@R‐PLT remained stable over a prolonged period, releasing only a small amount of drug during the observation period.

Collectively, these findings indicate that OE‐ACAD11@R‐PLT is capable of preferential accumulation within HCC tumor tissues and executing precise, microenvironment‐responsive drug release.

### 3.5 In Vitro Results of OE‐ACAD11@R‐PLT Induced Pyroptosis

Building upon the established role of ACAD11 in triggering pyroptosis and the intrinsic ability of R‐PLT to target tumors, we proceeded to unravel the molecular mechanisms through which OE‐ACAD11@R‐PLT induces pyroptosis in vitro. The study specifically focused on the NLRP3/Caspase‐1/GSDMD signaling pathway (Figure 5A). Time‐course morphological observation further revealed progressive pyroptosis‐associated changes, with increasingly evident cellular swelling and balloon‐like membrane protrusions in the OE‐ACAD11@PLT and, more prominently, OE‐ACAD11@R‐PLT groups at later time points (Figure S25). Ultrastructural changes in the cells were visualized by TEM. Cells in the Control and Vector@R‐PLT groups maintained an intact morphology with clear and well‐defined mitochondrial structures. In contrast, MHCC97H cells treated with OE‐ACAD11@R‐PLT exhibited characteristic morphological features of pyroptosis, including cellular swelling, plasma membrane rupture with pore formation (indicated by red arrows), leakage of cytosolic contents, and extensive vacuolization (Figure 5B). Consistently, analysis of the culture supernatants showed significantly increased levels of LDH, IL‐1β, and IL‐18 in the OE‐ACAD11@PLT and OE‐ACAD11@R‐PLT groups, with the highest levels observed in the OE‐ACAD11@R‐PLT group (Figure S26), further supporting the induction of pyroptosis.

**FIGURE 5:**
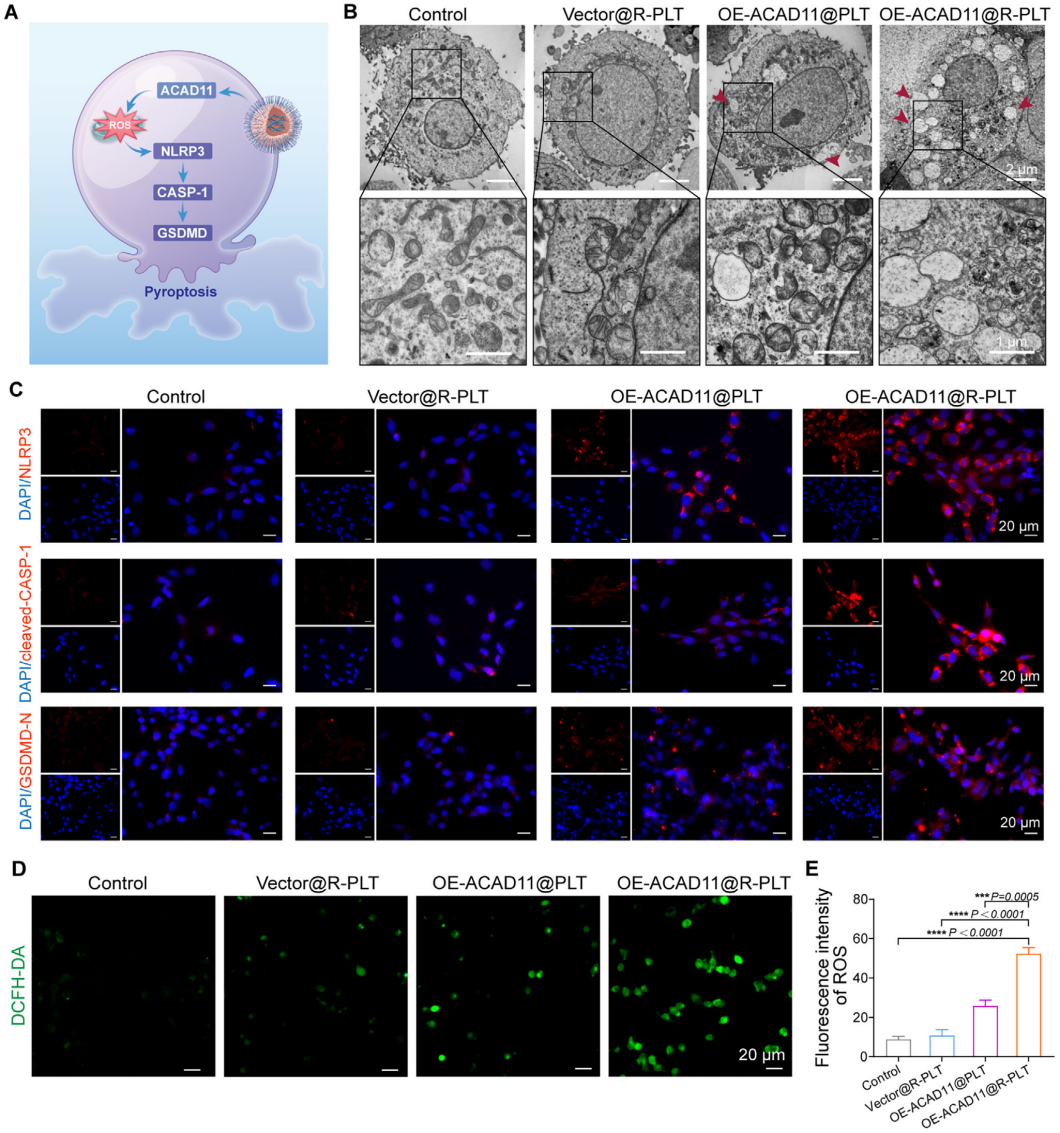
The in vitro pyroptosis induction of OE‐ACAD11@R‐PLT. (A) The schematic diagram of pyroptosis induced by OE‐ACAD11@R‐PLT via the NLRP3/Caspase‐1/GSDMD signaling pathway. (B) TEM images of MHCC97H cells. Red arrows indicate characteristic pyroptotic changes including cell swelling, membrane rupture, and vacuolization. (C) ICC images of pyroptosis‐related proteins (NLRP3, cleaved‐Caspase‐1, and GSDMD‐N) in MHCC97H cells. Blue, DAPI. Red, target proteins. (D) Intracellular ROS levels detected by DCFH‐DA staining (green fluorescence). (E) The quantitative analysis of ROS fluorescence intensity. Data are presented as mean ± SD (*n* = 3). Statistical significance was assessed using one‐way ANOVA with multiple comparisons. *** P < 0.001 and **** P < 0.0001.

A marked increase in NLRP3 intensity in the OE‐ACAD11@R‐PLT group was observed by immunocytochemistry (ICC). Consistent with downstream pathway activation, significant upregulation and membrane/perinuclear aggregation of Cleaved‐Caspase‐1 and GSDMD‐N were observed, indicating the formation of pyroptotic pores (Figure 5C). Semi‐quantitative fluorescence analysis further confirmed these marked differences (Figure S27). To further evaluate the changes in pyroptosis‐related proteins, Western blot analysis was performed to detect both the total and cleaved forms of CASP‐1 and GSDMD. Increased levels of total CASP‐1 and GSDMD, together with cleaved CASP‐1 and GSDMD‐N, were observed following OE‐ACAD11 treatment, with more pronounced changes in the OE‐ACAD11@R‐PLT group (Figure S28), further supporting activation of the CASP‐1/GSDMD‐mediated pyroptotic pathway.

Consistent with mitochondrial dysfunction, OE‐ACAD11@R‐PLT treatment triggered substantial ROS accumulation (Figure 5D,E) accompanied by a collapse in mitochondrial membrane potential. The latter was confirmed by JC‐1 staining, which showed a distinct reduction in the red/green fluorescence ratio (Figure S29).

Collectively, these findings demonstrate that OE‐ACAD11@R‐PLT effectively induces pyroptosis in MHCC97H cells in vitro by disrupting mitochondrial function to trigger a ROS burst, which subsequently activates the NLRP3/Caspase‐1/GSDMD signaling cascade.

### 3.6 In Vivo Therapeutic Efficacy of OE‐ACAD11@R‐PLT

Based on these promising in vitro results, we assessed the in vivo therapeutic efficacy of OE‐ACAD11@R‐PLT via the orthotopic HCC mouse model. Orthotopic HCC mice were randomized into four groups: Control, Vector@R‐PLT, OE‐ACAD11@PLT, and OE‐ACAD11@R‐PLT. Treatments were administered every 3 days starting from day 6 (Figure 6A). On day 28, mice were either sacrificed for tissue analysis (*n* = 3) or monitored for survival (n = 6). As shown in Figure 6B, mice in the Control, Vector@R‐PLT, and OE‐ACAD11@PLT groups exhibited mean survival times of 35.2 ± 1.6, 35.5 ± 2.34, and 40.0 ± 3.10 days, respectively. In contrast, the OE‐ACAD11@R‐PLT group achieved a significantly prolonged lifespan, with a mean survival time extending to 47.2 ± 3.19 days, which was significantly longer than that of the OE‐ACAD11@PLT group (*P* = 0.0042).

**FIGURE 6:**
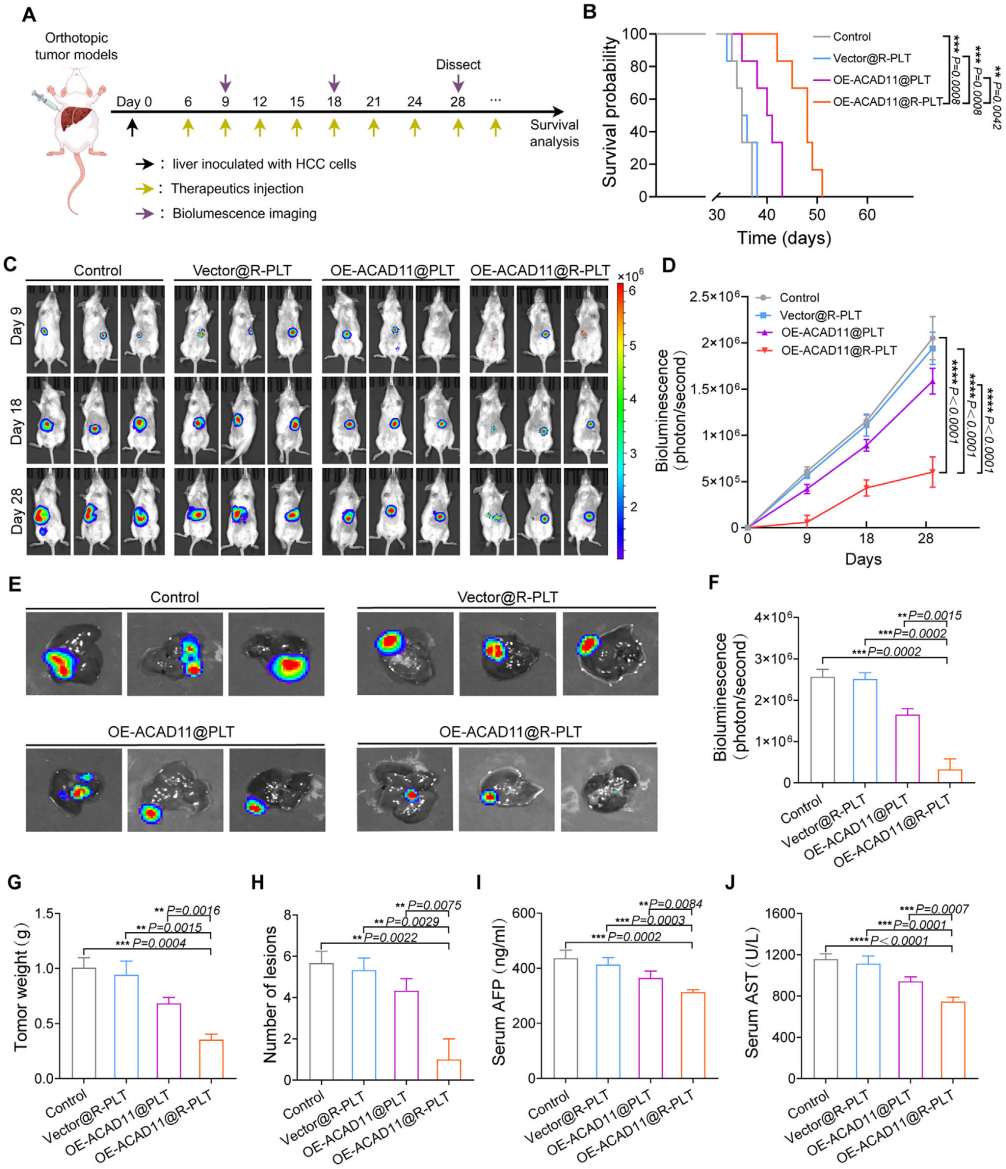
In vivo therapeutic efficacy of OE‐ACAD11@R‐PLT. (A) Illustration of drug administration timeline in orthotopic HCC mice. (B) KM curves of orthotopic HCC mice in the specified treatment groups. (C) Representative in vivo bioluminescence images of mice at days 9, 18, and 28. (D) Quantitative analysis of the bioluminescence signals in vivo (*n* = 6). (E) Representative ex vivo bioluminescence images of dissected livers. (F) Quantification of the bioluminescence intensity in the dissected livers. (G–J) Quantitative analysis of tumor weight (G), number of tumor nodules (H), serum AFP levels (I), and serum AST levels (J) in different groups. Data are presented as mean ± SD (*n* = 3). Statistical significance was assessed using two‐way ANOVA for longitudinal bioluminescence data and one‐way ANOVA with multiple comparisons for endpoint comparisons. Survival differences were analyzed using the log‐rank test. ** P < 0.01, *** P < 0.001 and **** P < 0.0001.

To visually monitor tumor progression in vivo, bioluminescence imaging was conducted at designated time points (days 9, 18, and 28). Compared to the other three groups, the OE‐ACAD11@R‐PLT group displayed the lowest bioluminescence intensity at all time points (Figure 6C), a finding confirmed by semi‐quantitative analysis (Figure 6D). Consistent with these in vivo findings, ex vivo imaging of excised livers on day 28 showed the weakest fluorescence signal in the OE‐ACAD11@R‐PLT group (Figure 6E,F). Furthermore, this group exhibited the lowest tumor weight and the fewest visible tumor nodules among all cohorts (Figure 6G,H).

A marked reduction in the expression of proliferation and metastasis markers (Bcl‐2, Ki‐67, MMP‐2, and PCNA) was demonstrated in the OE‐ACAD11@R‐PLT group by IHC analysis (Figure S30). Consistent with the observed tumor inhibition, serum AFP levels were markedly reduced in the OE‐ACAD11@R‐PLT group compared to controls (Figure 6I). Compared to the severe hepatic damage in control groups, evident from elevated AST, ALT, and ALP, the treatment group displayed significantly reduced levels of these markers (Figure 6J, Figure S31). These findings demonstrate that the targeted system not only eradicates tumors via pyroptosis but also mitigates liver injury without systemic toxicity, demonstrating excellent therapeutic safety and hepatoprotective potential.

### 3.7 In Vivo Immunotherapy Efficacy of OE‐ACAD11@R‐PLT

To unravel the underlying mechanism of the antitumor efficacy of OE‐ACAD11@R‐PLT, tumor tissues from orthotopic HCC mice were analyzed. Western blot analysis showed that ACAD11 expression was significantly upregulated in the OE‐ACAD11@R‐PLT group compared to the Control and Vector@R‐PLT groups, confirming effective targeted gene delivery and enrichment (Figure 7A). Further quantitative analyses showed that ACAD11 expression was significantly increased at both the mRNA and protein levels in the OE‐ACAD11@R‐PLT group (Figure S32). Notably, this ACAD11 upregulation triggered the activation of the NLRP3 inflammasome pathway, evidenced by substantially increased levels of NLRP3, cleaved Caspase‐1, and the pyroptosis executioner protein GSDMD‐N (Figure 7A). These results were substantiated by IHC analysis, where the OE‐ACAD11@R‐PLT group displayed the most pronounced immunoreactivity for pyroptosis‐associated proteins (Figure 7B).

**FIGURE 7:**
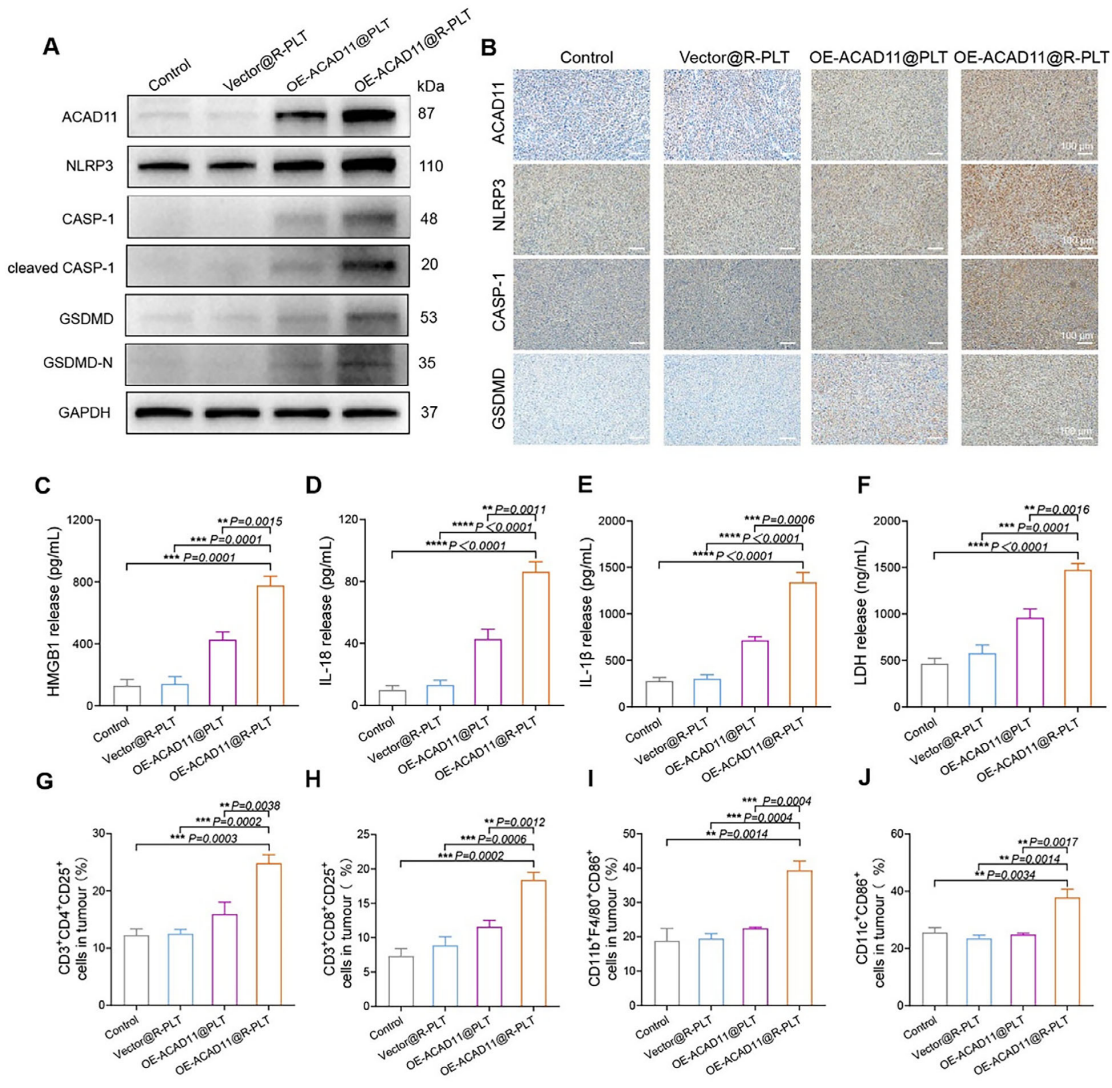
Schematic representation of in vivo pyroptosis induction mechanisms and immune microenvironment remodeling. (A) Western blot analysis of ACAD11, NLRP3, Caspase‐1 (CASP‐1), cleaved CASP‐1, GSDMD, and GSDMD‐N expression in tumor tissues across different treatment groups. (B) Representative IHC images of ACAD11, NLRP3, CASP‐1, and GSDMD in tumor sections. (C–F) Quantitative analysis of HMGB1 (C), IL‐18 (D), IL‐1β (E), and lactate dehydrogenase (LDH) (F) levels in tumor tissues measured by ELISA. (G–J) Flow cytometric quantification of tumor‐infiltrating immune cell populations, expressed as percentages of total CD45^+^ leukocytes: CD3^+^CD4^+^CD25^+^ T cells (G), CD3^+^CD8^+^CD25^+^ T cells (H), CD11b^+^F4/80^+^CD86^+^ pro‐inflammatory macrophages (I), and CD11c^+^CD86^+^ mature dendritic cells (J). Data are presented as mean ± SD (*n* = 3). Statistical significance was assessed using one‐way ANOVA with multiple comparisons. ** *P* < 0.01, *** *P* < 0.001, **** *P* < 0.0001.

As pyroptosis is an immunogenic cell death (ICD) characterized by membrane rupture, we further assessed the release of intracellular substances by ELISA. As depicted in Figure 7F, the OE‐ACAD11@R‐PLT group exhibited significantly increased lactate dehydrogenase (LDH) release, indicating compromised cell membrane integrity. Concurrently, the release of DAMPs such as HMGB1 (Figure 7C) markedly increased, alongside elevated pro‐inflammatory cytokines IL‐18 (Figure 7D) and IL‐1β (Figure 7E). These findings demonstrate that OE‐ACAD11@R‐PLT not only triggers tumor cell death but also successfully remodels the immunosuppressive tumor microenvironment into a highly immunogenic state.

We hypothesised that this pro‐inflammatory environment would reactivate the anti‐tumor immune response. Flow cytometric analysis revealed that OE‐ACAD11@R‐PLT treatment significantly promoted the maturation and infiltration of antigen‐presenting cells, characterised by increased proportions of mature dendritic cells (CD11c^+^CD86^+^, Figure 7J) and pro‐inflammatory macrophages (CD11b^+^F4/80^+^CD86^+^, Figure 7I). This induced a markedly enhanced adaptive immune response, manifested by maximal infiltration of CD3^+^CD4^+^ helper T cells (Figure 7G) and CD3^+^CD8^+^ cytotoxic T cells (Figure 7H) within tumor tissues. Representative gating strategies are provided in Figure S33. To further distinguish the contribution of CD8^+^ T‐cell‐mediated antitumor immunity from the direct tumor‐inhibitory effect of ACAD11, an in vitro co‐culture assay was performed using CD8^+^ T cells and MHCC97H cells. OE‐ACAD11 alone significantly inhibited MHCC97H cell growth, whereas co‐culture with CD8^+^ T cells further enhanced this inhibitory effect compared with OE‐ACAD11 treatment alone (Figure S34). These results provide functional evidence that CD8^+^ T‐cell‐mediated immune activity contributes additional antitumor effects beyond the direct tumor inhibition induced by ACAD11.

Collectively, these data indicate that OE‐ACAD11@R‐PLT initiates an ACAD11‐mediated pyroptotic cascade through the NLRP3/Caspase‐1/GSDMD pathway, thereby promoting antitumor immune activation and enhancing the overall therapeutic response.

### 3.8 In Vivo Biosafety of OE‐ACAD11@R‐PLT

We demonstrated in previous experiments that OE‐ACAD11@R‐PLT caused no significant hepatic toxicity. Building on this observation, we proceeded to evaluate its systemic in vivo biocompatibility and potential side effects. Representative visceral organ, including the heart, spleen, lungs, and kidneys, were harvested from orthotopic HCC mice on days 9, 18, and 28 for H&E staining. Histological analysis revealed no apparent pathological abnormalities or tissue damage in these organs compared to the pre‐treatment state (Figure S35A).

Concurrently, peripheral blood samples were collected to assess physiological function via serum biomarker analysis. Throughout the treatment course, peripheral blood samples were harvested at scheduled intervals for the assessment of serum biochemical parameters:TP and ALB reflecting systemic nutritional status, LDH and CK reflecting myocardial injury, and BUN and Cr reflecting renal function. As illustrated in Figure S35B–G, the levels of these biomarkers remained within the physiological range, with no discernible statistical deviations from the baseline values.

Furthermore, consistent with these biochemical findings, the dynamic physiological status of the mice was evaluated by meticulously monitoring relative body weight changes in both the short‐term mechanistic and long‐term survival cohorts (Figure S36A,B). While mice in the Control and Vector@R‐PLT groups experienced rapid and progressive weight loss indicative of severe tumor burden and cancer cachexia, and the non‐targeted OE‐ACAD11@PLT group still exhibited a moderate weight decrease, the systemic administration of OE‐ACAD11@R‐PLT effectively prevented tumor‐induced weight loss. Remarkably, mice in this targeted treatment group maintained healthy, steady weight gain throughout the extended 51‐day observation period. This sustained physiological stability firmly substantiates the excellent long‐term biosafety and in vivo tolerability of our therapeutic system.

Collectively, these findings substantiate the high safety margin of OE‐ACAD11@R‐PLT, as evidenced by the absence of significant off‐target toxicity or damage to vital tissues within the experimental dosage window. This confirms that the therapeutic system is well‐tolerated and exhibits negligible systemic toxicity in vivo.

## 4 Discussion

Therapeutic outcomes in HCC are severely constrained by the tumor's inherent resistance to apoptosis and its highly immunosuppressive tumor microenvironment [39, 40]. Consequently, conventional therapeutic strategies designed to induce apoptosis often struggle to produce sustained efficacy. In this context, pyroptosis, a lytic form of regulated cell death mediated by gasdermin proteins, has emerged as a novel strategy to overcome therapeutic resistance due to its ability to release abundant pro‐inflammatory cytokines and DAMPs, thereby remodeling the immune microenvironment [14]. Du et al. recently demonstrated that inducing pyroptosis in HCC via a smart nano vaccine releases significant amounts of DAMPs and activates the cGAS‐STING pathway, effectively reversing the immunosuppressive TME [41]. Furthermore, Hou et al. indicated that utilizing a molecular switch to convert cell death modalities from apoptosis to pyroptosis is critical for promoting tumor necrosis and anti‐tumor immunity [42]. Inspired by these findings, this study aims to explore whether this process can be triggered in HCC through specific metabolic regulation.

Herein, we identify ACAD11 as a critical regulator of ROS‐NLRP3‐mediated pyroptosis in HCC. Beyond its canonical role in fatty acid oxidation, ACAD11 overexpression was found to significantly disrupt mitochondrial homeostasis [43]. Specifically, this upregulation provokes a surge in mitochondrial reactive oxygen species (mtROS), which subsequently triggers NLRP3 inflammasome assembly and GSDMD cleavage. By disrupting mitochondrial homeostasis via this mtROS‐dependent axis, the process not only induces cell death but also activates innate immunity [44, 45]. Li et al. revealed that modulating mitochondrial calcium regulators dictates drug resistance in HCC [46]. However, despite their promise, strategies relying on exogenous pharmacological inducers face significant limitations [47]. Conventional chemotherapeutics or small‐molecule metabolic inhibitors often lack tumor specificity, leading to systemic toxicity [48]. In contrast, our study demonstrates that directly manipulating the levels of the endogenous enzyme ACAD11 functions as an intrinsic switch to initiate NLRP3‐mediated pyroptosis, offering a novel, mechanism‐driven avenue for inducing pyroptosis in HCC.

Despite identifying a potent target, achieving precision delivery remains a challenge [49]. Platelets are widely used as carriers due to their natural tumor‐homing capabilities [50]; however, their application in HCC requires careful consideration of the liver's unique pathological background [51]. HCC frequently arises on a foundation of chronic hepatitis or cirrhosis, creating a sustained inflammatory microenvironment that alters biodistribution profiles [52]. As demonstrated by Zhang et al., platelets exhibit intrinsic tropism toward inflammatory sites, a behavior driven by their interactions with activated endothelium and inflammatory cytokines [23]. However, in HCC, this inflammatory tropism poses a hurdle. Diffuse hepatic inflammation functions as a ‘sink,’ non‐specifically sequestering platelets and limiting their accumulation in the tumor core [53]. Biodistribution analysis revealed extensive accumulation of unmodified platelets in inflamed liver tissue. This underscores the limitations of passive homing and necessitates active targeting strategies to minimize background retention.

Surface engineering with cRGDyk was employed to minimize off‐target retention. This modification replaces weak P‐selectin interactions with high‐affinity binding to integrin α_v_β_3_ thereby enhancing tumor accumulation [54]. Xu et al. confirmed that this cyclic peptide significantly facilitates rapid receptor‐mediated endocytosis in integrin‐overexpressing tumor cells [27]. Given that the expression level of integrin α_v_β_3_ on HCC cells is significantly higher than that on background hepatocytes, the OE‐ACAD11@R‐PLT system effectively discriminates between malignant lesions and the inflamed background [35]. Synergizing the immune evasion of platelet membranes with cRGDyk targeting, the system maximizes intracellular ACAD11 delivery to trigger the pyroptotic cascade. Overall, this study uncovers the ACAD11‐pyroptosis axis and presents a robust bioengineered nanoplatform for precision HCC therapy.

Several limitations of the present study should also be acknowledged. Although the CD8^+^ T‐cell co‐culture experiments support an additional immune‐mediated antitumor effect beyond the direct tumor‐inhibitory activity of ACAD11, the relative contribution of this immune component to the overall therapeutic efficacy remains to be quantitatively defined in vivo. Future studies employing in vivo CD8^+^ T‐cell depletion will therefore be important to further clarify this contribution.

Despite its efficacy in remodeling the TME, the OE‐ACAD11@R‐PLT system faces translational challenges regarding human immune heterogeneity and potential immunogenicity, which may not be fully recapitulated in murine models. In addition, the hematological and immunological risks associated with systemic platelet‐based administration should be carefully considered. Premature or unintended platelet activation may generate a procoagulant platelet phenotype, including phosphatidylserine exposure, which provides a catalytic surface for thrombin generation and fibrin formation and may thereby promote coagulation activation and thrombus formation [55, 56]. Therefore, the potential thrombogenic risks associated with repeated administration warrant further investigation. Although no overt systemic toxicity was observed in the present study, dedicated assessments of platelet activation, coagulation parameters, and thrombotic events will be required to comprehensively establish the long‐term safety of this delivery platform. Consequently, establishing GMP‐compliant production protocols and validating long‐term safety in more clinically relevant models will be essential prerequisites for clinical translation.

## 5 Conclusion

Herein, the engineered platelet nanoplatform represents a feasible approach to deliver the metabolic regulator ACAD11 to HCC, demonstrating superior antitumor efficacy compared to conventional strategies. The cRGDyk‐modified carrier successfully circumvented non‐specific retention in the inflamed liver background, facilitating the active targeting of ACAD11 to malignant lesions. Upon internalization, the payload triggered the NLRP3/Caspase‐1/GSDMD signaling cascade, initiating robust pyroptosis. This approach minimized the systemic toxicity associated with exogenous chemotherapeutics and enhanced therapeutic efficacy by effectively reversing the highly immunosuppressive tumor microenvironment. Through mechanism‐driven intervention, OE‐ACAD11@R‐PLT significantly inhibited tumor progression and activated antitumor immunity. Collectively, our study offers fresh insights into the development of metabolism‐based nanomedicines for HCC.

## Author Contributions

**Hongyi Bao**: Writing – original draft, Validation, Software, Methodology, Investigation, Data curation. **Wei Feng**: Writing – original draft, Data curation, Investigation. **Wei Zong**: Writing – review & editing, Methodology, Data curation. **Yi Li**: Writing – review & editing, Software, Formal analysis. **Xinyue Qin**: Software, Methodology. **Xinyue Wang**: Investigation. **Yubo Yang**: Investigation. **Guanhua Chen**: Data curation. **Yiting Hou**: Software. **Xuan Tan**: Software. **Lin Chen**: Writing – review & editing, Project administration, Funding acquisition, Conceptualization. **Ming Zhang**: Writing – review & editing, Supervision, Resources, Conceptualization. **Feng Wang**: Writing – review & editing, Supervision, Project administration, Funding acquisition, Conceptualization.

## Ethics Statement

The retrospective use of archived human pathological tissue and blood samples was approved by the Ethics Committee of the Affiliated Hospital of Nantong University (approval no. 2024‐L004), and written informed consent was obtained from all participants. All animal experiments were approved by the Animal Ethics Committee of Nantong University (approval no. P20240124‐010). Clinical Study Registration: Not applicable. This study was not a prospective interventional clinical trial and therefore did not require registration.

## Conflicts of Interest

The authors declare no conflicts of interest.

## Supporting information




**Supporting File**: advs78219‐sup‐0001‐SuppMat.docx.

## Data Availability

The data that support the findings of this study are available from the corresponding author upon reasonable request.
