# Supplementary material for: A Biomimetic cRGDyk‐Engineered Platelet Nanoplatform for ACAD11 Delivery to Trigger Pyroptosis in Hepatocellular Carcinoma
Source: Adv Sci (Weinh). 2026 Oct 8:e78219. Online ahead of print. doi: 10.1002/advs.78219 (PMC13650033; doi:10.1002/advs.78219)
Supplement: Supplementary file 1 [file ADVS-9999-e78219-s001.docx]

**Supplementary Information**

**A Biomimetic cRGDyk-Engineered Platelet Nanoplatform for ACAD11 Delivery to Trigger Pyroptosis in Hepatocellular Carcinoma**

Hongyi Bao^a,b,1^, Wei Feng^a,b,1^, Wei Zong^b,1^, Yi Li^b,1^, Xinyue Qin^b^, Xinyue Wang^b^, Yubo Yang^b^, Guanhua Chen^b^, Yiting Hou^b^, Xuan Tan^b^, Lin Chen^c*^, Ming Zhang^d*^, Feng Wang^a,b*^

a. Department of Laboratory Medicine, The Afﬁliated Wuxi People’s Hospital of Nanjing Medical University, Wuxi People’s Hospital, Wuxi Medical Center, Nanjing Medical University, Wuxi 214023, China

b. Research Center of Clinical Medicine, Affiliated Hospital of Nantong University, Medical School of Nantong University, Nantong 226001, China

c. Nantong Institute of Liver Diseases, Nantong Third People's Hospital Affiliated Nantong Hospital 3 of Nantong University, Nantong 226006, China

d. Department of Laboratory Medicine, Changzhou Maternal and Child Health Care Hospital, Changzhou 213000, China

Hongyi Bao, Wei Feng, Wei Zong and Yi Li Contributed equally to this work.

* Correspondence:

xiaobei227@sina.com (Lin Chen);

zmzw0701@163.com (Ming Zhang)

richardwangf@163.com (Feng Wang)

Figure S1. Kaplan-Meier survival analysis of candidate genes based on the TCGA database. Survival curves displaying the 5-year overall survival probabilities of HCC patients stratified by low (black) and high (red) expression levels of ACAD11, HIGD1B, PHYHD1, and TTC9. Survival differences were assessed using the log-rank test. Hazard ratios (HR) and log-rank P-values are indicated for each gene. Results reveal that high expression of ACAD11 exhibits the most significant correlation with prognosis, while PHYHD1 shows moderate significance, and HIGD1B and TTC9 display no statistical significance. ** P* < 0.05*, ** P* < 0.01*, *** P* < 0.001 and ***** P* < 0.0001. ns, nonsignificant (*P* > 0.05).

Figure S2. Immunohistochemical staining of ACAD11 in HCC tissues and adjacent non-cancerous tissues. The adjacent non-cancerous tissues exhibit strong positive staining (brown), whereas the HCC tissues show significantly downregulated ACAD11 expression. Scale bar: 100 μm.

Figure S3. The effects of OE-ACAD11 and si-ACAD11 on the proliferation of HCC cells were analyzed by CCK-8 Proliferation Assay. The growth curves showed that ACAD11 overexpression significantly inhibited the proliferation of MHCC97H and Huh7 cells, while ACAD11 knockdown promoted the proliferation of HCCLM3 cells. Data are presented as mean ± SD (n = 3). **** P* < 0.001 and ***** P* < 0.0001; two-way ANOVA.

Figure S4. The effects of OE-ACAD11 and si-ACAD11 on HCC cell migration were analyzed by wound healing. Representative images (left) and quantitative analysis of the wound closure percentage (right) indicated that ACAD11 overexpression significantly impaired the migration of MHCC97H and Huh7 cells, whereas ACAD11 silencing enhanced the migration of HCCLM3 cells. Data are presented as mean ± SD (n = 3). *** P* < 0.01*,* and **** P* < 0.001; *t*-test.

Figure S5. The effects of siRNA-ACAD11 on HCC cell migration and invasion were analyzed by Transwell assay. ACAD11 knockdown significantly enhanced the migratory and invasive potential of HCC cells compared to the control. Data are presented as mean ± SD (n = 3). *** P* < 0.01*,* and **** P* < 0.001; *t*-test. Scale bar: 50 μm.

Figure S6. Time-course morphological characterization of MHCC97H cells following OE-ACAD11 transfection. Representative microscopic images of MHCC97H cells at 0, 6, 12, and 24 h after OE-ACAD11 transfection. Progressive pyroptosis-associated morphological changes were observed over time, including cellular swelling and characteristic balloon-like membrane protrusions, which became evident at 12 h and more pronounced at 24 h. Red arrows indicate representative pyroptotic morphological features. Scale bar: 20 μm.

Figure S7. Quantitative analysis of pyroptosis-associated factor release following ACAD11 overexpression. The levels of LDH, IL-1β, and IL-18 in the culture supernatants of Vector- and OE-ACAD11-transfected MHCC97H cells were quantified. ACAD11 overexpression significantly increased the extracellular levels of LDH, IL-1β, and IL-18 compared with the Vector group, further supporting the induction of pyroptosis. Data are presented as mean ± SD (n = 3). ***P* < 0.01, ****P* < 0.001, and *****P* < 0.0001; *t*-test.

Figure S8. The GO enrichment of top 5 terms categorized into biological processes (BP), cellular components (CC), and molecular functions (MF) between Control and OE-ACAD11 groups. The red outline highlighted the significant enrichment of the "regulation of response to stimulus" pathway within the BP category.

Figure S9. Quantitative analysis of intracellular ROS levels. Treatment with OE-ACAD11 resulted in a significant elevation in intracellular ROS accumulation compared to the control group. *** *P* < 0.001; *t*-test.

Figure S10. Quantitative analysis of the JC-1 aggregate/monomer ratio. Statistical analysis of the red-to-green fluorescence ratio indicated a significant loss of mitochondrial membrane potential in ACAD11-overexpressing cells compared to the control. Data are presented as mean ± SD (n = 3). ** *P* < 0.005; *t*-test.

Figure S11. NAC attenuated ACAD11 overexpression-induced ROS accumulation. (A) Representative fluorescence images of intracellular ROS detected by DCFH-DA staining in Vector, OE-ACAD11, and OE-ACAD11 + NAC-treated MHCC97H cells. Scale bar, 50 μm. (B) Quantitative analysis of ROS fluorescence intensity. ACAD11 overexpression markedly increased intracellular ROS levels, whereas NAC treatment significantly attenuated ROS accumulation. Data are presented as mean ± SD (n = 3). *****P*< 0.0001; ns, nonsignificant (*P* > 0.05); one-way ANOVA with multiple comparisons.

Figure S12. Effects of NAC on pyroptosis-related proteins following ACAD11 overexpression. Representative Western blot analysis of ACAD11, CASP-1, cleaved CASP-1, GSDMD, and GSDMD-N in Vector, OE-ACAD11, and OE-ACAD11 + NAC-treated MHCC97H cells. GAPDH was used as the loading control.

Figure S13. Evaluation of the CASP-3/GSDME pathway following ACAD11 overexpression. Representative Western blot analysis of ACAD11, NLRP3, CASP-3, cleaved CASP-3, GSDME, and GSDME-N in MHCC97H cells transfected with Vector or OE-ACAD11. ACAD11 overexpression increased NLRP3 expression, whereas no evident increase in cleaved CASP-3 or GSDME-N was observed. GAPDH was used as the loading control.

Figure S14. IHC staining images of ACAD11 in orthotopic HCC tumor tissues following treatment with traditional viral vectors encoding OE-ACAD11. Compared to the control group, the OE-ACAD11 group displayed no significant increase in ACAD11 protein expression levels, indicating the limited delivery efficiency and poor tumor accumulation of conventional viral vectors in the liver tumor microenvironment. Scale bar: 100 μm.

Figure S15. DNase protection assay of OE-ACAD11 plasmid encapsulated in R-PLTs. Plasmid-specific qPCR showed that DNase I markedly reduced naked OE-ACAD11 plasmid DNA, whereas the plasmid signal was largely preserved in intact OE-ACAD11@R-PLTs but significantly decreased after platelet lysis, indicating that OE-ACAD11 was predominantly encapsulated within the platelets rather than surface-adsorbed. Data are presented as mean ± SD (n = 3). *****P* < 0.0001; ns, not significant; one-way ANOVA with multiple comparisons.

Figure S16. Functional activity of OE-ACAD11 plasmid released from activated platelets. The released plasmid was collected from activated OE-ACAD11@R-PLTs and subsequently transfected into MHCC97H cells. qRT-PCR analysis showed that the released OE-ACAD11 plasmid significantly increased ACAD11 mRNA expression compared with the released Vector plasmid, while no significant difference was observed between the released OE-ACAD11 plasmid and freshly prepared OE-ACAD11 plasmid, indicating that the released plasmid retained its transfection activity after platelet loading and release. Data are presented as mean ± SD (n = 3). ****P < 0.0001; ns, not significant; one-way ANOVA with multiple comparisons.

Figure S17. The particle size of OE-ACAD11@R-PLT was monitored over a multi-day observation period. Results indicated that the hydrodynamic diameter remained stable throughout the study, particularly during the first 6 days, suggesting that the platelet delivery system remained in a resting state without spontaneous activation. Data are presented as mean ± SD (n = 3).

Figure S18. Flow cytometric analysis of cRGDyk surface modification on platelets. (A) Representative flow cytometric histograms of FITC fluorescence in PLTs and cRGDyk-modified platelets (R-PLTs). (B) Median fluorescence intensity (MFI) of FITC-labeled cRGDyk in PLTs and R-PLTs. Data are presented as mean ± SD (n = 3). ****P* = 0.0001; *t*-test.

Figure S19. Gross images of excised tumor-bearing livers corresponding to the ex vivo fluorescence images in Figure 4A. Gross images of excised livers from individual orthotopic HCC-bearing mice treated with OE-ACAD11@PLT or OE-ACAD11@R-PLT are shown in the same order as the corresponding ex vivo fluorescence images in Figure 4A. Grossly identifiable tumor lesions are outlined by black circles to clarify the anatomical localization of the tumor regions used for fluorescence analysis.

Figure S20. Quantitative analysis of fluorescence intensity in the liver region of orthotopic HCC mice. Comparative analysis demonstrated that the OE-ACAD11@R-PLT group exhibited significantly higher accumulation compared to the OE-ACAD11@PLT group, indicating superior tumor-targeting efficiency and enrichment capability. Data are presented as mean ± SD (n = 3). ***** P* < 0.0001; *t*-test.

Figure S21. *Ex vivo* biodistribution analysis and tumor accumulation assessment. Major organs and tumors were collected 24h post-administration for *ex vivo* fluorescence imaging. Representative images (left) and quantitative analysis of fluorescence intensity (right) revealed that the OE-ACAD11@R-PLT group exhibited significantly enhanced accumulation in tumor tissues compared to the OE-ACAD11@PLT group. The total fluorescence intensity in the tumor was markedly higher in the OE-ACAD11@R-PLT group, indicating that the R-PLT modification effectively improved specific targeting capabilities and prolonged *in vivo* residence time. Data are presented as mean ± SD (n = 3). ** P* < 0.05 and **** P* < 0.001. ns, nonsignificant (*P* > 0.05); *t*-test.

Figure S22. Schematic illustration of the in vitro tumor-targeting specificity assay using a Transwell system. QSG-7701 cells and MHCC97H cells were cultured in the lower chamber, which was separated from the upper chamber by a Transwell insert containing a 0.4 μm microporous membrane. OE-ACAD11@R-PLT was added to the upper chamber. Following a 24 h incubation, the Transwell inserts were removed, and the adhesion of OE-ACAD11@R-PLT in the vicinity of the underlying cells was observed via scanning electron microscopy (SEM).

Figure S23. Immunofluorescence detection of P-selectin expression on OE-ACAD11@R-PLT before and after activation. Representative images showed that P-selectin (red) was minimally expressed in the unactivated group but was significantly upregulated on OE-ACAD11@R-PLT following activation. Scale bar: 100 μm.

Figure S24. Particle size distribution analysis of components released from OE-ACAD11@R-PLT. The results exhibited distinct peaks at 82 nm and 1152 nm. This distribution reflected the rapid release of the internal payload from OE-ACAD11@R-PLT following activation.

Figure S25. Time-course morphological changes of MHCC97H cells following different treatments. Representative microscopic images of MHCC97H cells in the Control, Vector@R-PLT, OE-ACAD11@PLT, and OE-ACAD11@R-PLT groups at 0, 6, 12, and 24 h. Progressive pyroptosis-associated morphological changes, including cellular swelling and balloon-like membrane protrusions, were observed in the OE-ACAD11@PLT and, more prominently, OE-ACAD11@R-PLT groups over time, whereas the Control and Vector@R-PLT groups showed relatively stable morphology. Red arrows indicate representative balloon-like membrane protrusions. Scale bar: 20 μm.

Figure S26. Quantitative analysis of pyroptosis-associated factor release. The levels of LDH, IL-1β, and IL-18 in the culture supernatants were quantified across different treatment groups. The results demonstrated that the release of LDH, IL-1β, and IL-18 was significantly increased in the OE-ACAD11@R-PLT group compared with the Control, Vector@R-PLT, and OE-ACAD11@PLT groups, further supporting the induction of pyroptosis. Data are presented as mean ± SD (n = 3). ***P* < 0.01, ****P* < 0.001, and *****P* < 0.0001; one-way ANOVA with multiple comparisons.

Figure S27. Semi-quantitative immunofluorescence analysis of pyroptosis-related markers. The integrated fluorescence signals of NLRP3, cleaved CASP-1, and GSDMD-N were quantified across different treatment groups. The results demonstrated that the fluorescence intensities of these markers were significantly upregulated in the OE-ACAD11@R-PLT group compared to the Control, Vector@R-PLT, and OE-ACAD11@PLT groups, indicating robust activation of the pyroptotic pathway. Data are presented as mean ± SD (n = 3). **** P* < 0.001 and ***** P* < 0.0001; one-way ANOVA with multiple comparisons.

Figure S28. Western blot analysis of pyroptosis-related proteins in MHCC97H cells. Protein levels of ACAD11, CASP-1, cleaved CASP-1, GSDMD, and GSDMD-N were detected in MHCC97H cells following the indicated treatments. GAPDH was used as the loading control.

Figure S29. Assessment of mitochondrial membrane potential changes using JC-1 staining. Representative fluorescence images displayed the alterations in mitochondrial membrane potential. Red fluorescence represented JC-1 aggregates (indicating high membrane potential), while green fluorescence indicated JC-1 monomers (indicating low membrane potential). The OE-ACAD11@R-PLT group exhibited a significant shift from red to green fluorescence compared to the Control and Vector@R-PLT groups. Quantitative analysis of the aggregate/monomer (red/green) ratio confirmed a marked reduction in mitochondrial membrane potential in cells treated with OE-ACAD11@R-PLT, suggesting severe mitochondrial dysfunction. *** P* < 0.01*,* and **** P* < 0.001; one-way ANOVA with multiple comparisons. Scale bar: 20 μm.

Figure S30. Immunohistochemical analysis of proliferation and metastasis-related markers in tumor tissues. Representative immunohistochemical images displayed the expression levels of Bcl-2, Ki-67, MMP-2, and PCNA in different treatment groups. The Control and Vector@R-PLT groups exhibited strong positive staining (brown) for all markers. The OE-ACAD11@PLT group showed reduced expression of these markers compared to the control group; however, the OE-ACAD11@R-PLT group demonstrated the most significant downregulation, indicating that it exerted the most significant inhibition on proliferation and metastasis. Scale bar: 20 μm.

Figure S31. Measurement of serum ALT and ALP levels. Compared to the other groups, the serum levels of ALT and ALP in the OE-ACAD11@R-PLT group were significantly reduced. These results indicated that OE-ACAD11@R-PLT treatment effectively alleviated liver injury and protected liver function. Data are presented as mean ± SD (n = 3). *** P* < 0.01*, *** P* < 0.001 and ***** P* < 0.0001; one-way ANOVA with multiple comparisons.

Figure S32. Quantitative analysis of ACAD11 expression in tumor tissues. (A) qRT-PCR analysis of ACAD11 mRNA expression in tumor tissues from the indicated treatment groups. (B) Densitometric analysis of ACAD11 protein expression based on Western blot results. ACAD11 protein levels were normalized to GAPDH and expressed relative to the Control group. Data are presented as mean ± SD (n = 3). ****P* < 0.001, and *****P* < 0.0001; one-way ANOVA with multiple comparisons.

Figure S33. Representative gating strategy for flow cytometric analysis of tumor-infiltrating immune cells. (A) Sequential gating of lymphocytes based on FSC-A/SSC-A, single cells based on SSC-H/SSC-W, live cells by exclusion of FVD-positive cells, and CD45⁺ leukocytes. The CD45⁺ population was used as the common parent population for subsequent immune cell subset analysis. (B) Gating of CD3⁺CD4⁺ T cells followed by identification of CD3⁺CD4⁺CD25⁺ T cells. (C) Gating of CD3⁺CD8⁺ T cells followed by identification of CD3⁺CD8⁺CD25⁺ T cells. (D) Gating of CD11b⁺F4/80⁺ macrophages followed by identification of CD11b⁺F4/80⁺CD86⁺ pro-inflammatory macrophages. (E) Gating of CD11c⁺CD86⁺ mature dendritic cells. Numbers shown within the gates indicate the percentages of cells within the respective parent populations.

Figure S34. CD8⁺ T-cell co-culture assay distinguishes immune-mediated antitumor activity from the direct tumor-inhibitory effect of ACAD11. (A) Representative flow cytometric gating strategy for fluorescence-activated sorting of CD3⁺CD8⁺ T cells from peripheral blood-derived lymphocytes. Lymphocytes were first gated based on FSC-A and SSC-A, followed by single-cell selection and identification of the CD3⁺CD8⁺ population. (B) Representative microscopic images of non-activated and activated CD8⁺ T cells. Activated CD8⁺ T cells exhibited prominent cellular aggregation. Scale bar, 100 μm. (C) Growth curves of MHCC97H cells in the Vector, Vector + CD8⁺ T cell, OE-ACAD11, and OE-ACAD11 + CD8⁺ T cell groups. OE-ACAD11 alone inhibited MHCC97H cell growth, whereas co-culture with CD8⁺ T cells further enhanced this inhibitory effect. Data are presented as mean ± SD (n = 3). *****P* < 0.0001; two-way ANOVA.

Figure S35. *In vivo* biosafety evaluation of OE-ACAD11@R-PLT. (A) Representative H&E staining images of major organs (heart, spleen, lungs, and kidneys) collected from tumor-bearing mice at days 0, 9, 18, and 28 from various treatment groups. No noticeable histological damage or inflammatory lesions were observed. Serological biochemical analysis of peripheral blood samples from mice at the indicated time points. The levels of (B) total protein (TP) and (C) albumin (ALB) were measured to assess nutritional status and hepatic function. (D) Lactate dehydrogenase (LDH) and (E) creatine kinase (CK) levels were quantified to evaluate myocardial integrity. (F) Blood urea nitrogen (BUN) and (G) creatinine (Cr) levels were determined to monitor renal function. Data are presented as mean ± SD (n = 3). ns, nonsignificant (*P* > 0.05); one-way ANOVA with multiple comparisons. Scale bar: 100 μm.

Figure S36. *In vivo* systemic biosafety and body weight monitoring of orthotopic HCC-bearing mice. (A) Relative body weight changes of the mechanistic evaluation cohort over the 28-day experimental period. Data are presented as mean ± SD (n = 3 per group). (B) Long-term relative body weight changes of the independent survival observation cohort (corresponding to the Kaplan-Meier analysis in Figure 6B) over the 51-day period. The initial sample size was n = 6 per group. Note: The sample size in S36B dynamically decreased over time due to disease progression and tumor-related mortality; lines for specific groups terminate when all animals in that cohort perished. Statistical analysis was performed using body weight data collected up to Day 36 (the last time point when mice in the Control group were alive). ***** P* < 0.0001. ns, nonsignificant (*P* > 0.05); two-way ANOVA.

**Table. S1.** **Correlation analysis between ACAD11 expression level and clinicopathological characteristics of HCC.**

| **Characteristic** | **Total(n)** | | **ACAD11(n%)**  **Low (n=25) High(n=25)** | | ***P*** |
| --- | --- | --- | --- | --- | --- |
| **Gender** |  | |  |  |  |
| Male | 42 | | 21 (50.0%) | 21 (50.0%) | 1.000 |
| Female | 8 | | 4 (50.0%) | 4 (50.0%) |  |
| **Age (years)** |  | |  |  |  |
| ≤60 | 41 | | 22 (53.7%) | 19 (46.3%) | 0.269 |
| ＞60 | 9 | | 3 (33.3%) | 6 (66.7%) |  |
| **Tumor Size (cm)** |  | |  |  |  |
| ≤5 | 25 | | 6 (24.0%) | 19 (76.0%) | ＜0.01** |
| ＞5 | 25 | | 19 (76.0%) | 6 (24.0%) |  |
| **Histological differentiation** |  | |  |  |  |
| Well+Moderate | 35 | | 14 (40.0%) | 21 (60.0%) | 0.064 |
| Poor | 15 | | 11 (73.3%) | 4 (26.7%) |  |
| **Grade** |  | |  |  |  |
| Ⅰ+Ⅱ | 29 | | 9 (31%) | 20 (69%) | 0.002** |
| Ⅲ+Ⅳ | 21 | | 16 (76.2%) | 5 (23.8%) |  |
| **T stage** |  | |  |  |  |
| Ⅰ+Ⅱ | 26 | | 8 (30.8%) | 18 (69.2%) | 0.005** |
| Ⅲ+Ⅳ | 24 | | 17 (70.8%) | 7 (29.2%) |  |
| **TNM stage** |  | |  |  |  |
| Ⅰ+Ⅱ | 28 | | 9 (32.1%) | 19 (67.9%) | 0.004** |
| Ⅲ+Ⅳ | 22 | | 16 (72.7%) | 6 (27.3%) |  |
| **Serum AFP (ng/ml)** | |  |  |  |  |
| ＞200 | | 35 | 18 (51.4%) | 17 (48.6%) | 0.758 |
| ≤200 | | 15 | 7 (46.7%) | 8 (53.3%) |  |
| **HBV infection** |  | |  |  |  |
| Positive | 41 | | 20 (48.8%) | 21 (51.2%) | 1.000 |
| Negative | 9 | | 5 (55.6%) | 4 (44.4%) |  |

**Table S2. Sequence information for primers used in this study.**

| **Gene** | **Sequence (5’-3’)** |
| --- | --- |
| **ACAD11** | F: TCCCCGTTCCCAAGCCTATA |
| **HIGD1B**  **PHYHD1**  **TTC9** | R: GGAAGATTCGACCCTGCACA  F: CTGAGGTCTCGTGGTTCCAC  R: CTGTGCCATCCTCTTGACGT  F: AAGTGCTGGGATTACAGGCC  R: CAGGCCCAAGATACTTGGCA  F: GTCTGGTGTGGCCTTCTACC  R: TTGGTTGTTGGGTCCTTGCT |

**Table S3. Sequence information for the plasmid used in this study.**

| **Plasmid** | | **Sequence (5’-3’)** |
| --- | --- | --- |
| **pLenti-CMV-ACAD11-PGK-Puro-WPRE** | F: CGCAAATGGGCGGTAGGCGTG  R: GGATGTGGAATGTGTGCGAGGC | |

**Table S4. Sequence information for siRNA vectors used in this study.**

| **siRNA** | **Sequence (5’-3’)** |
| --- | --- |
| **si-ACAD11-1** | F: GGAUAGCUUUCAAGAAGAATT |
| **si-ACAD11-2**  **si-ACAD11-3**  **si-NC** | R: UUCUUCUUGAAAGCUAUCCTT  F: GGACAGUUCCAAUGAUAAATT  R: UUUAUCAUUGGAACUGUCCTT  F: GGCAGAAACUGGACUACAATT  R: UUGUAGUCCAGUUUCUGCCTT  F: UUCUCCGAACGUGUCACGUTT  R: ACGUGACACGUUCGGAGAATT |
